# Thin and Flexible Breeze-Sense Generators for Non-Contact Haptic Feedback in Virtual Reality

**DOI:** 10.1007/s40820-025-01670-y

**Published:** 2025-02-13

**Authors:** Kaijun Zhang, Zhe Liu, Yexi Zhou, Zhaoyang Li, Dazhe Zhao, Xiao Guan, Tianjun Lan, Yanting Gong, Bingpu Zhou, Junwen Zhong

**Affiliations:** 1 Department of Electromechanical Engineering and Centre for Artificial Intelligence and Robotics, University of Macau, Macau SAR, 999078 People’s Republic of China; 2 Joint Key Laboratory of the Ministry of Education, Institute of Applied Physics and Materials Engineering, University of Macau, Macau SAR, 999078 People’s Republic of China

**Keywords:** Virtual reality, Wearable electronics, Non-contact haptic feedback, Breeze-sense, Piezoelectret actuator

## Abstract

In the realm of virtual reality (VR), haptic feedback is integral to enhance the immersive experience; yet, existing wearable devices predominantly rely on skin contact feedback, lacking options for compact and non-contact breeze-sense feedback. Herein, we propose a compact and non-contact working model piezoelectret actuator for providing a gentle and safe breeze sensation. This easy-fabricated and flexible breeze-sense generator with thickness around 1 mm generates air flow pressure up to ~ 163 Pa, which is significantly sensed by human skin. In a typical demonstration, the breeze-sense generators array showcases its versatility by employing multiple coded modes for non-contact information transmitting. The thin thinness and good flexibility facilitate seamless integration with wearable VR setups, and the wearable arrays empower volunteers to precisely perceive the continuous and sudden breeze senses in the virtual environments. This work is expected to inspire developing new haptic feedback devices that play pivotal roles in human–machine interfaces for VR applications.

**Supplementary Information:**

The online version contains supplementary material available at 10.1007/s40820-025-01670-y.

## Highlights

- The breeze-sense generators generate significant air flow pressure output of ~ 163 Pa that can easily be sensed by human skin and have an overall thickness around 1 mm.
- Volunteers successfully identify multiple programming patterns transmitted by the generators array.
- A wearable breeze-sense feedback system effectively provides the continuous or sudden breeze senses in virtual reality environments.

**Supplementary Information:**

The online version contains supplementary material available at 10.1007/s40820-025-01670-y.

## Graphical abstract

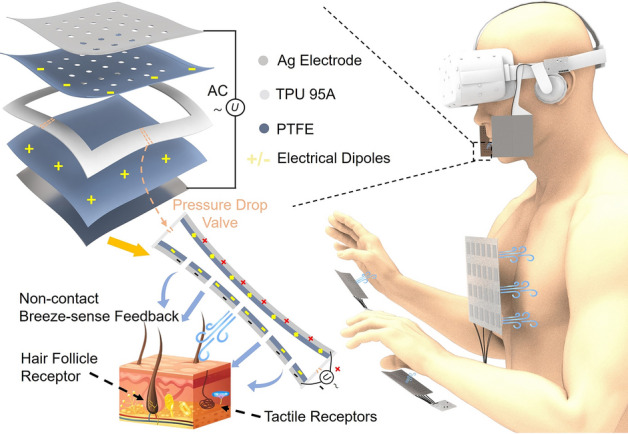

## Introduction

In recent years, human–machine interfaces (HMIs) have become integral windows facilitating communication between humans and the digital world, particularly in virtual reality (VR), augmented reality (AR), and mixed reality (MR) [1–4]. The utilization of flexible sensors with exceptional performances has been widespread for monitoring signals from the human body and the external environments [5–14]. Concurrently, the advanced information feedback interfaces, such as AR/VR glasses for delivering visual and auditory information, have played crucial roles in realizing closed-loop HMIs [15, 16]. Furthermore, the skin, as the largest sensory organ, has emerged as the preferred platform for embedding feedback interfaces in HMIs [17, 18]. Previous efforts have devised numerous skin-based HMIs incorporating haptic feedback, significantly enhancing the user experience. This has led to a heightened demand for diverse feedback experiences in AR/VR [19–21]. In specific, researchers focus on developing wearable devices that can provide multiple haptic feedback sensations and more realistic effects [22]. In the pursuit of user immersion in HMIs, attributes such as thinness, lightweight construction, and ergonomic wearability have become paramount. Recently, the majority of wearable feedback devices combined in VR mostly demand skin contact. Typically, these skin-contacting haptic feedback devices include the following methods: electromagnetic technology [23, 24, 37], shape memory alloys [25], piezoelectric devices [26, 27], piezoelectret devices [28–30], dielectric elastomers [31, 32], and pneumatically actuated polymer structures [33, 34]. Combing with other senses feedback, good experiences are provided for users in VR scenarios. For instance, Yu et al. integrated visual, auditory, tactile, and olfactory senses with AR/VR to provide users with a multisensory experience [35–38]. Lee et al. employed vibration and Joule heat to enhance realism in the metaverse [39].

However, as users demand deeper immersion when using VR, skin comfort has become new demands for wearable feedback devices. Therefore, researchers try to develop non-contact feedback devices [40, 41]. These non-contact feedback devices achieve a more comfortable and safer experience for users. For example, ultrasonic feedback devices allow users to feel objects in VR without touching devices [40]. Another possible source of non-contact haptic stimulus is air flow. In fact, the breeze-sense is a very common tactile sensation in daily life, and the integration of a non-contact breeze-sense feedback device into a wearable HMI system is a valuable addition to current haptic feedback technology. Previously, electric fans, pneumatic actuation, and piezoelectric fans are used, so that users can feel the wind in the virtual sceneries [42–46]. This indicates that non-contact breeze-sense feedback in the VR environments is an effective way to increase the user’s immersive experience. Nevertheless, these devices are usually not available as wearable feedback devices because of their large weight, hardness, and volume. Consequently, there is still a lack of a breeze-sense generator that is compact, safe, and easy to integrate with VR devices.

In this work, we propose an innovative strategy to the challenges faced by non-contact feedback devices in VR applications—a thin and flexible breeze-sense generator. When AC voltage is applied to the sandwich-structured breeze-sense generator with inside cavity, the device produces *Z*-directional volume compression and recovery by means of electrostatic force, pushing the inside air to generate a breeze that passes through the holes of upper layer to produce non-contact haptic feedback for human skin. Key advancements of this work include: (i) a breeze-sense generator has a thickness around 1 mm and great flexibility to maintain more than 50% of the airflow pressure output when bent at 50°; (ii) during continuous 5-h working, the device maintains air flow pressure output at ~ 163 Pa, affirming the excellent mechanical durability; (iii) volunteers successfully identify multiple programming patterns transmitted by the generators array; and (iv) a wearable breeze-sense feedback system effectively provides the continuous or sudden breeze senses in VR environments.

## Experimental Section

### Fabrication of the Breeze-Sense Generator

The polytetrafluoroethylene (PTFE) films are purchased from Jincheng Plastic. The conductive silver painting is purchased from Shenzhen Jingzhe technology. The detail fabrication steps are shown in Fig. S1. Step I-cutting PTFE: A laser cutter (mingchang 4060, 70 W) is used to cut PTFE with a thickness of 100 µm into squares with designed sizes; Step II-electrode manufacturing: one surface of PTFE films with different sizes is painted with silver paint, and the thickness of silver is approximately 10–15 µm; Step III-Corona charging: Corona charging method is used to generate negative and positive electrostatic charges on the PTFE surface without electrode, as shown in Fig. S2, the PTFE surface is faced to the corona charge needle tip with the distance of 5 cm, the negative charging voltage generated by a high-voltage source (Dongwen DW-N503-4ACD2) is − 17 kV, the positive charging voltage generated by a high-voltage source (Dongwen DW-P503-4ACD2) is + 23 kV, the charging time is 5 min for each sample; Step IV-printing Support: a 3D printer (Bambu Lab X1) is used to print flexible support frame of 95A TPU with designed thickness; Step V-assembling breeze-sense generators: the two PTFE/Ag films that have been positively and negatively polarized are glued to the 3D printed frame by double sided tape.

### Fabrication of the PVDF Sensor

PVDF piezoelectric film coated with silver is purchased from PolyK with a thickness of 12 µm. The main body of PVDF sensor consists of a PLA-printed support and a PVDF piezoelectric membrane.

### COMSOL Multiphysics Simulation

We simulate laminar flow-displacement responses using 3D finite element analysis in COMSOL Multiphysics. In the model, a stable state solution is first calculated for fully coupled solid mechanics and laminar flow. Then, the displacement is defined in the model with sine function. The simulation of the generator output valves in laminar flow is extracted by reading the pressure distribution on a plane 1 cm away from the generator. In specific, Table S1 shows the geometrical structure parameters of the breeze-sense generator in the COMSOL simulation. Table S2 provides the mechanical characterization of materials in the COMSOL simulation.

### Portable Circuits

The driving signal generator, voltage amplifier, and decoding circuit are the three components that make up the decoding and driving circuit. A pulse-width modulation (PWM) scheme is generated by the ICM7555 chip and controlled by low-pass filter. The DRV2700 chip amplifies PWM signals by varying the feedback resistance and capacitance, resulting in high-voltage signals that are sent to the solid-state relays. These signals are then utilized to regulate the generators' on/off state based on the decoded data.

### Characterizations

1. The surface potential of the samples is tested with an electrostatic voltmeter (Trek 347).
2. The outputs of the PVDF sensor are calibrated by an electromechanical output testing system. In this system, the outputs are measured by a NI USB 6341 data acquisition system, and regular mechanical stimulation applied on the samples is provided by a Modal shaker (JZK-10, Sinocera Piezotronics, Inc. China) controlled by a YE1311 (Sinocera Piezotronics, China) sweep signal generator and a YE5872A (Sinocera Piezotronics, China) power amplifier.
3. The driving signals are generated by a signal generator source (Keysight 33210A, Tektronix Corporation, America) and amplified by an amplifier (E-464 HVPZT AMPLIFIER, Physical Instrument GmbH & Co. KG, Karlsruhe, Germany).
4. The vibrating displacement of the devices is measured by a LDV (Vibro One, Polytec), with a range of 500 µm, a sensitivity of 250 µm V^−1^.
5. The SEM images are probed by a high-resolution field emission scanning electron microscope (Sigma FE-SEM, Zeiss Corporation).
6. A multifunctional noise analyzer (AWA6228 + , AIHUA) is used to test the sound pressure level of device during operation.
7. A thermal imaging camera (HIKVISION HM-TPH11-3AXF) is used to record the temperature change of a working device.
8. The study protocol is thoroughly reviewed and approved by the Ethical Committee of University of Macau (approval number BSERE21-APP022-FST). Informed consents are signed by volunteers prior to their participation in this study.

## Results and Discussion

### Design Strategy

**Fig. 1:**
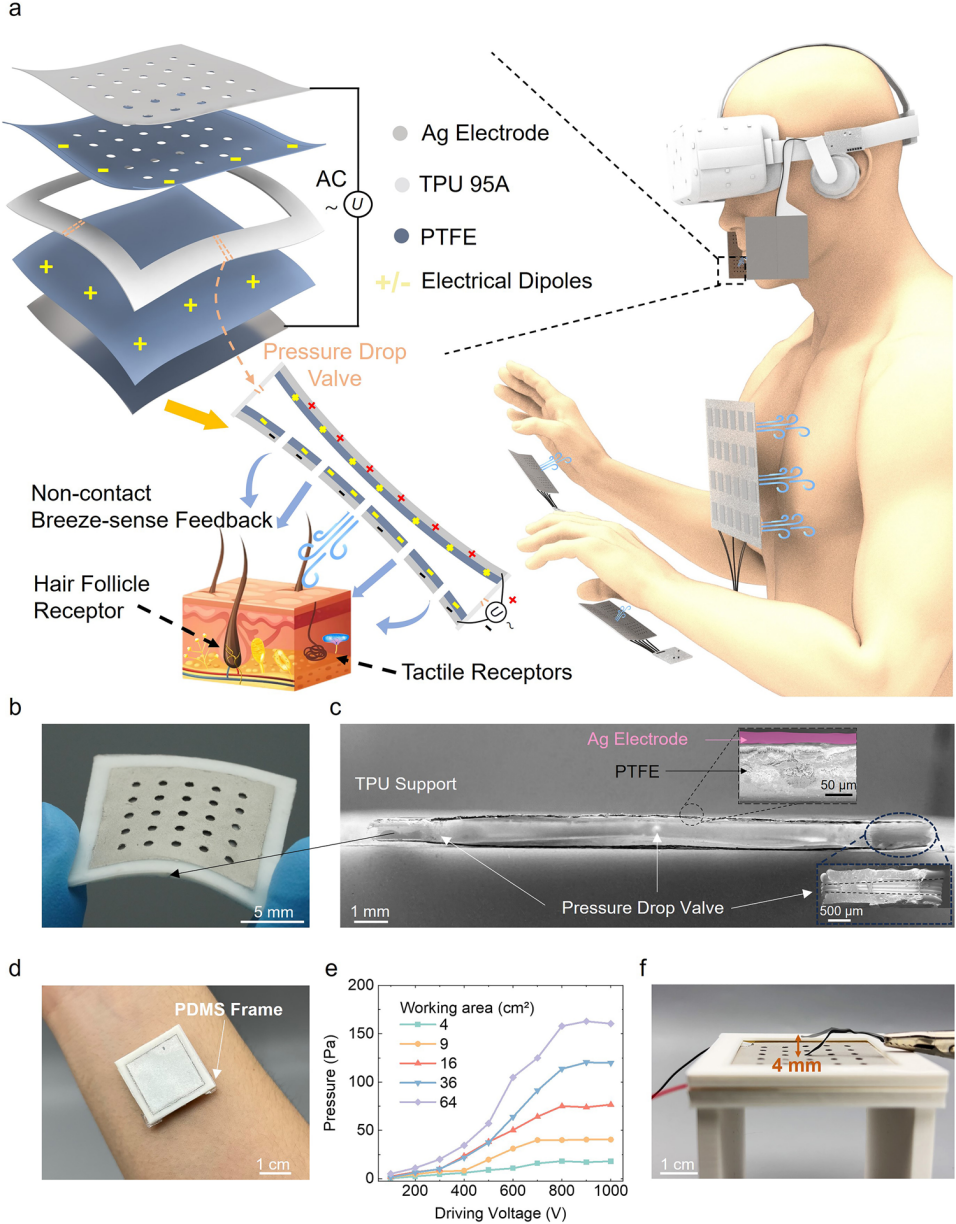
Concept and design strategy. **a** Schematic illustrating the proposed wearable breeze-sense feedback system, right part shows using breeze-sense generators for providing feedback for multiple parts of human body, and left part shows the detailed structure and working mechanism of the breeze-sense generator. **b** An image showing the breeze-sense generator with a working area of 2 × 2 cm^2^. **c** Cross-sectional SEM imaged of a breeze-sense generator. **d** An image showing a 2 × 2 cm^2^ breeze-sense generator when be worn on human arm. **e** Recorded by a commercial PVDF sensor, the air flow pressure outputs produced by devices with different working areas and driving voltages, each device works at resonant frequency and the measuring distance is 1 cm. **f** Image showing a 4 × 4 cm^2^ breeze-sense generator driven by an AC voltage (*V*_*P*–*P*_ = 500 V, frequency = 160 Hz) for blowing a piece of paper up to height of 4 mm

The schematic of the proposed wearable breeze-sense feedback system is illustrated in Fig. 1a. In specific, when a user faces a scenario with breeze existing in the VR environment, the breeze-sense generators will be activated, creating a gentle breeze that can be felt by the user. The breeze-sense generators can be integrated with a VR headset or textiles for generating feedback for multiple parts of human body. The sandwich structure of the breeze-sense generator, shown as the exploded view (upper left in Fig. 1a), consists of a negative charged top polytetrafluoroethylene (PTFE, 100 µm thick)/silver (Ag, 15–20 µm thick) layer with punched holes, a TPU 95A support with thickness of 0.75 mm in the middle for creating inside cavity, and a positive charged bottom PTFE/Ag layer, with detailed fabrication process as shown in Figs. S1 and S2. Figure 1b, c illustrates the image and cross-sectional scanning electron microscope (SEM) image of a flexible breeze-sense generator, and this 2 × 2 cm^2^ device has a thickness of ~ 970 µm and weight of ~ 0.45 g (Fig. S3). The breeze-sense generator is a piezoelectret actuator [27–30] (lower left in Fig. 1a). When driven by an AC voltage, the electrostatic force between the top and bottom PTFE/Ag layers produces *Z*-directional volume compression and recovery, and the electrical dipoles composed of inner positive and negative charges generated by Corona charging method have the equal effect of DC bias voltage, thus lowering the driving voltage [47]. After Corona charging, the surface potential values versus time curves of PTFE/Ag layers for 14 days are shown in Fig. S4, maintaining at + 1400 and -1800 V, respectively. The cyclical volume compression and recovery motions push the air inside the device to pass through the punched holes of the upper layer, and then, the generated air flow stimulates the tactile receptors [48, 49] and hair follicle receptors [50] to make the user feel the non-contact breeze-sense feedback. It should be noted that the non-contact working model of our device ensures the safety and comfort in wearable applications. Moreover, pressure drop valves across the four boundaries of TPU support are designed to reduce the negative pressure that hampers the recovery motion of the device (Fig. 1c). The schematic diagram of the one-way pressure drop valve is shown in Fig. S5 a, with parameters of 600 µm external diameter and 250 µm internal diameter. The fluid simulation results of the pressure drop valve are shown in Fig. S5b, which demonstrates that the pressure drop of the air flowing outward is much larger than the air flowing inward. An image of the device being worn on the arm is shown in Fig. 1d, with the PDMS frame for not allowing the device to contact with the skin or sweat to get into the device. The PDMS frame is also used in the devices array and a protective edge is also added to protect the user's safety (Fig. S6). Although the device is close to the skin, the vibrations do not generate heat or noise (Fig. S7).

A piezoelectric pressure sensor made of commercial PVDF with an area of 5 × 5 cm^2^ is used to measure the air flow pressure generated by the breeze-sense generators (Fig. S8). As illustrated in Fig. 1e, the air flow pressure outputs increase with the driving voltage and effective working area of the device, a maximum wind pressure of ~ 163 Pa is generated when the working area is 64 cm^2^, under driving voltage (*V*_*P*–*P*_) of 800 V, resonant frequency of 50 Hz, and measuring distance of 1 cm. It should be noted that the air flow pressure generated by normal breathing motions is below 150 Pa [51, 52]. As a result, the outputs of our breeze-sense generators can be easily sensed by human skin. Figure 1f vividly shows the generated air flow. Under driving *V*_*P*–*P*_ of 500 V and frequency of 160 Hz, a 4 × 4 cm^2^ device blow up a piece of paper for a height of ~ 4 mm (Movie S1). We should emphasize that the breeze-sense generator is used without skin contact, and the surface of the device close to the human skin is also grounded (high electrical potential is not applied on this surface). So, the device is very safe and will not cause harm to the human body.

### Performances Improvement

**Fig. 2:**
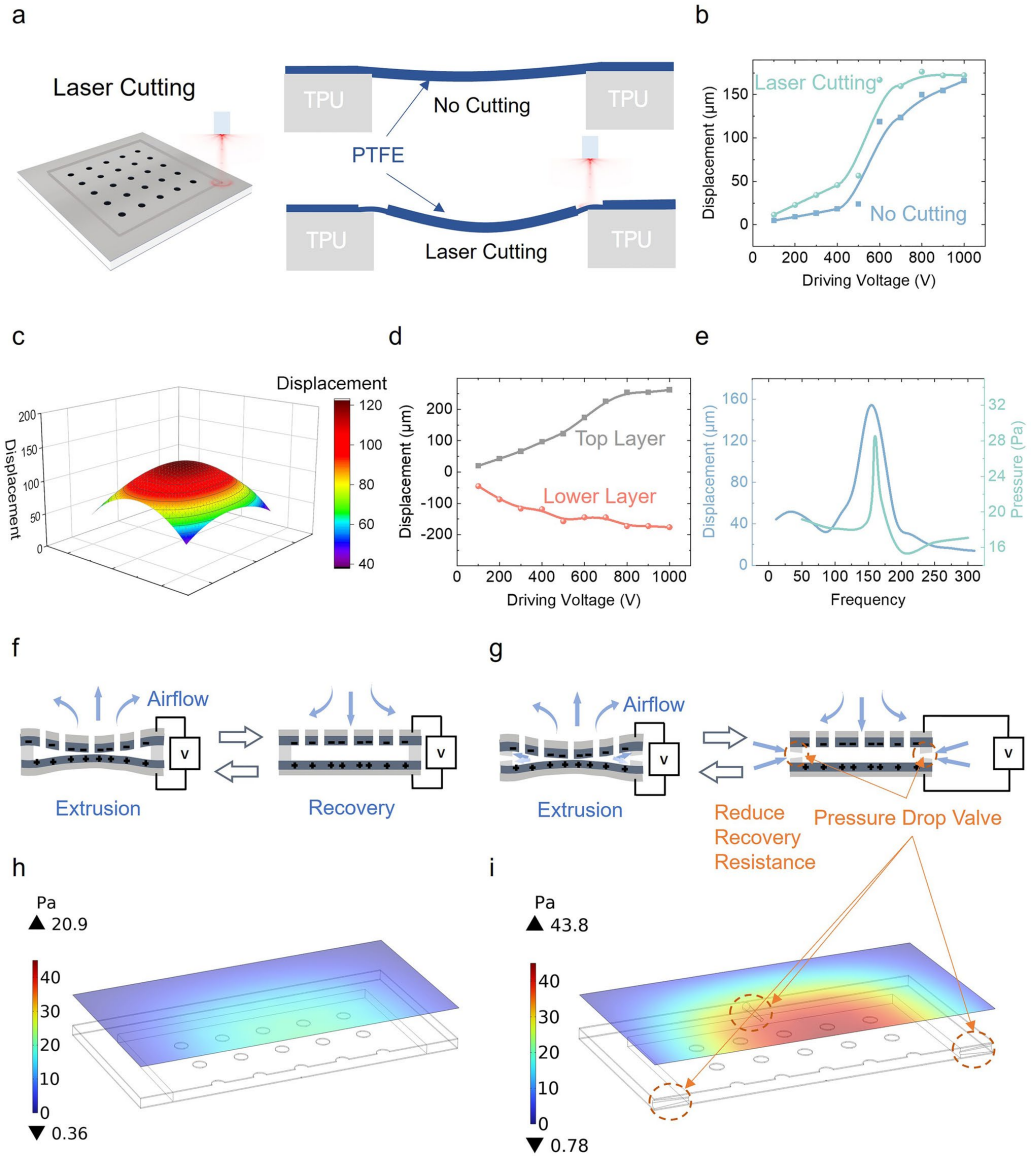
Performances improvement. **a** Schematic diagrams showing the laser cutting process for PTFE/Ag layer, and vibrations for laser cutting and no cutting cases. **b** Vibration displacements for PTFE/Ag layer with laser cutting and no cutting, at V_P-P_ of 100–1000 V and a fixed frequency of 160 Hz. **c** Vibration displacement distribution on the top layer of a 4 × 4 cm^2^ breeze-sense generator (*V*_*P*–*P*_ = 500 V, frequency = 160 Hz). **d** Vibration displacement at the centre of the top and lower layers of a 4 × 4 cm^2^ breeze-sense generator (*V*_*P*–*P*_ = 100–1000 V, frequency = 160 Hz). **e** Air flow pressure outputs and vibration displacements for a 4 × 4 cm^2^ breeze-sense generator (*V*_*P*–*P*_ = 500 V, frequency = 1–300 Hz) to indicate the resonant frequency. Schematic diagrams illustrating the compression and recovery process of breeze-sense generators **f** without and** g** with pressure drop valves. The simulation results show the pressure distribution on a plane at a distance of 1 cm away** h** before and **i** after the construction of pressure drop valves

The structure of breeze-sense generators is optimized for increasing air flow pressure output. Typically, at a fixed driving frequency, increasing the vibration amplitude of the top and bottom PTFE/Ag layer tends to effectively increase the air flow pressure output. Therefore, we use a 7.5 W laser to cut the boundaries where the PTFE and TPU are in contact, as shown in Fig. 2a. After cutting, there is a ~ 50 µm deep and ~ 50 µm wide defect in the PTFE boundaries (Fig. S9). The optical images of PTFE before and after laser cutting are also shown in Fig. S10. Compared to the untreated case, we obtain an average 35% increase in vibration amplitude after laser treatment, in which vibration displacements are measured a laser Doppler vibrometry (LDV), as shown in Fig. 2b. In specific, the vibration amplitude improvement under low driving voltage region is significant.

To further study the vibration of the device, scanning LDV is used to measure the entire vibrating layers of a 4 × 4 cm^2^ breeze-sense generator. Figure 2c shows the vibration displacements distribution of the top layer at frequency of 160 Hz and *V*_*P*–*P*_ of 500 V, and the maximum displacement occurs in the centre of the layer, which reaches ~ 125 µm. Driven by the same conditions, the maximum vibration displacement of the lower layer also occurs at the centre, as shown in Fig. S11. Figure 2d illustrates the maximum displacements for top and bottom layers at fixed frequency of 160 Hz and *V*_*P*–*P*_ from 100 to 1000 V. As the vibration resistance of the top layer with punched holes is smaller, it has larger vibration displacement than that of the bottom layer. The maximum displacements and air flow pressure outputs at fixed *V*_*P*–*P*_ of 500 V and frequency from 1 to 300 Hz is indicated in Fig. 2e. The device has maximum outputs at resonant frequency of 160 Hz, and we normally drive the devices with different working areas at their corresponding resonant frequency (Fig. S12) in the following measurements.

In fact, after the inner air is extruded from the breeze-sense generator, the internal pressure becomes smaller. This creates a significant vibration resistance to the recovery process of the device, as shown in Fig. 2f. Therefore, we propose to construct pressure drop valves across four boundaries of the TPU support, which can reduce the air inflow resistance and does not significantly reduce the flow rate of the air extrusion (Fig. 2g). For a more in-depth understanding of the operation and effect of the pressure drop valve, we develop a 3D multiphysics field finite element model. Figure S13 shows the vibration waveform of the top layer, which approximates a sine wave. Consequently, the pressure generated by laminar flow on the top layer is evaluated by using vibrational displacements in simulation model employing a fluid–solid coupling approach. Specifically, in the simulation, we input the top and bottom layer displacements (*V*_*P*–*P*_  = 500 V, frequency = 160 Hz) obtained from the LDV test. The details of the 3D model modelling dimensions are in Table S1, and the material properties for simulation are in Table S2. Figure 2h, i illustrates the pressure distribution in the planes at a distance of 1 cm away from the top layer of breeze-sense generator, before and after adding the pressure drop valves. Simulation results show that under the same driving conditions, the pressure drop valves across the boundaries of TPU support can efficiently enhance the air flow pressure outputs. The valves can increase the output of air flow pressure by ~ 100%.

### Key Parameters Characterizations

**Fig. 3:**
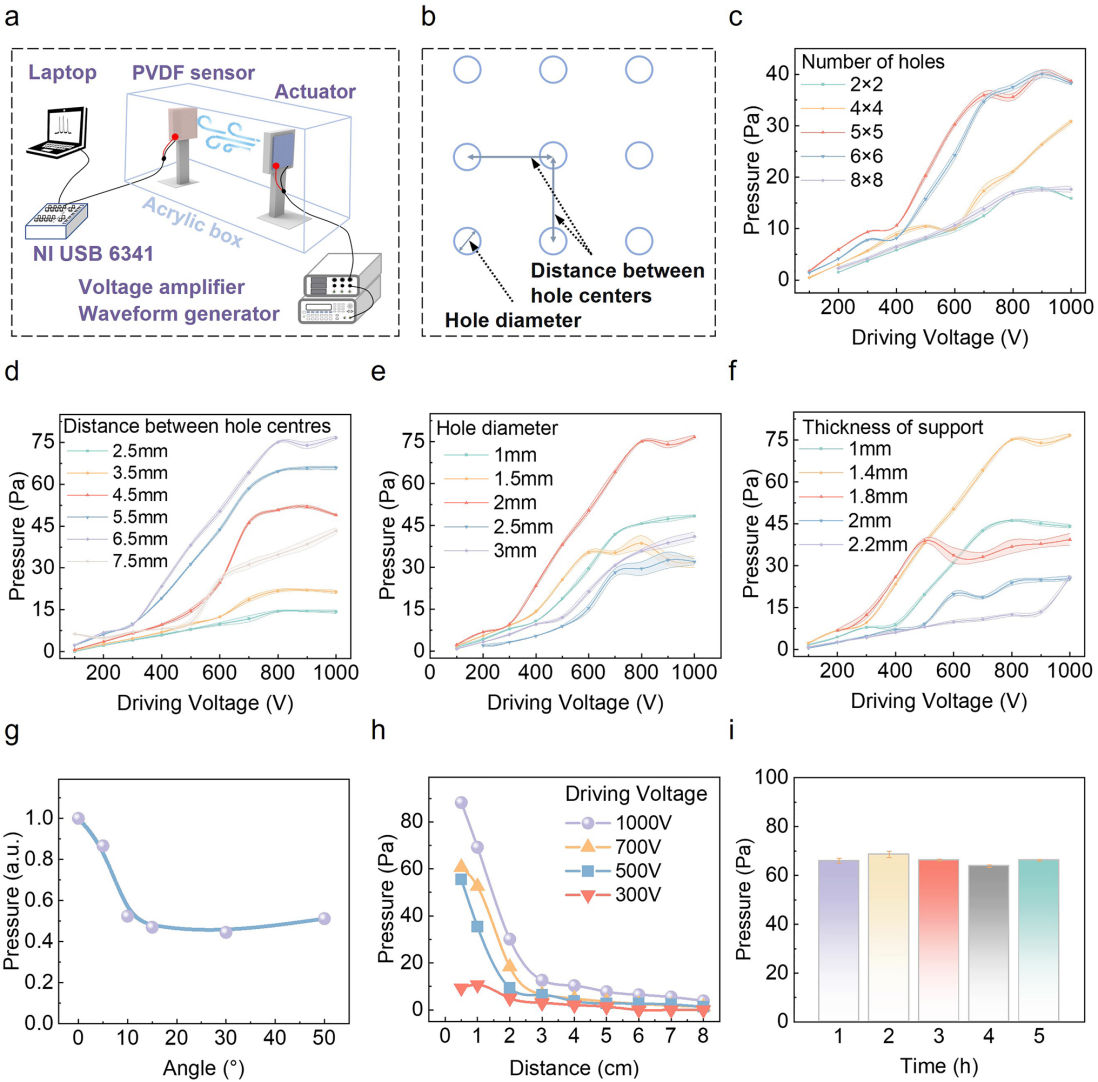
Key parameters characterizations. **a** Schematic illustration of the air flow pressure measuring setup. **b** Schematic diagram of hole diameter and the distance between hole centres. **c** Output pressure values of breeze-sense generators with various number of holes. **d** Output pressure values of breeze-sense generators when changing the distance between hole centres. **e** Output pressure values of breeze-sense generators when changing the hole diameter. **f** Output pressure values of breeze-sense generators when changing the thickness of the TPU support. **g** Normalized output pressure values when changing bending angles (*V*_*P*–*P*_ = 500 V). **h** Output pressure values *versus* the measuring distances. **i** Output pressure values during 5 h of continuous working time. (*V*_*P*–*P*_ = 500 V). Except as otherwise stated, the working area of a testing device is 4 × 4 cm^2^, the driving frequency is fixed at 160 Hz, and the driving voltage-V_P-P_ is from 100–1000 V

In the output testing (Fig. 3a), we connect the PVDF sensor to the NI date acquisition equipment after placing it at a suitable distance from the breeze-sense generator. Meanwhile, the driving conditions of breeze-sense generators are controlled by a waveform generator and a voltage amplifier. The optical image of the test environment is presented in Fig. S14, where a transparent acrylic box is employed to prevent other airflow interferences. Except as otherwise stated, the working area of a testing device is 4 × 4 cm^2^, the driving frequency is fixed at 160 Hz, and the driving voltage-*V*_*P*–*P*_ is from 100–1000 V.

Punched holes on the top layer have key effects on the output performances. Figure 3b indicates the diameter of the hole and the distance between hole centres we defined. The number of punched holes mainly affects the amount of charge stored on the PTFE electret film as the increasing punched holes will reduce the actual working area. As shown in Fig. 3c, the output pressure values of breeze-sense generators with various numbers of punched holes punched are tested, having a peak output pressure of ~ 40 Pa when the number of holes is 5 × 5 array with a hole diameter of 2 mm and the distance between hole centres is 2.5 mm (*V*_*P*–*P*_ = 900 V). Based on hole numbers of 5 × 5 array, we further optimize the output pressure by adjusting the distance between hole centres and the hole diameter. The distribution of holes in the PTFE/Ag layer affects the direction and volume of the extruded air flow during vibration. As the central area of the top layer has the maximum displacement (Fig. 2c), the over concentrated holes in the centre makes the most effective working area disappear and decrease the general vibration of the device, and then, the device cannot effectively compress the internal air cavity. The too scattered distribution of holes will prevent the extruded airflow from being concentrated, causing most of the energy to be consumed in surface turbulence, preventing the formation of effective laminar flow and further reducing performance. When the distance between hole centres is 6.5 mm and the hole diameter is 2 mm, the pressure output reaches maximum value of ~ 77 Pa, as indicated in Fig. 3d, e. Another key parameter is the thickness of the TPU support that forms the air cavity inside the device. When the TPU support is too thin, there is no enough air cavity space and the top and bottom PTFE/Ag layers will contact during working to make the positive and negative charges neutralize. If the TPU support is too thick, the electrostatic force will not be strong enough to generate large vibrating displacement. As illustrated in Fig. 3f, a maximum air flow pressure of ~ 77 Pa is obtained, when the TPU support thickness is 1.4 mm. It should be noted that the power consumption of our device is just ~ 4 mW when the driving *V*_*P*–*P*_ is 1000 V (Table S3).

Furthermore, to prove the good flexibility of the breeze-sense generator, we design brackets that can bend the device into different angles, as shown in Fig. S15. Figure 3g shows that the output air flow pressure is above 50% of the flat case, when the bending angel is up to 50°. The air flow pressure outputs decrease with the measuring distance. Figure 3h plots the pressure outputs for different distances and driving voltages. The distance between the device and the detector/volunteer is set as 1 cm to demonstrate that the breeze-sense is obviously detected/felt, which is reasonable for normal wearable conditions. Figure 3i presents the stability measurement of the breeze-sense generator for continues working of 5 h (*V*_*P*–*P*_  = 500 V, frequency = 160 Hz). The variation of outputs is less than 10%, indicating the excellent output stability that is critically important for the practicability. Moreover, the output pressure of the breeze-sense generator is not significantly affected by the normal humidity (< 70%RH) and temperature (20–50 °C), as shown in Fig. S16.

### Non-Contact Breeze-Sense Feedback for Coding Information Transfer

**Fig. 4:**
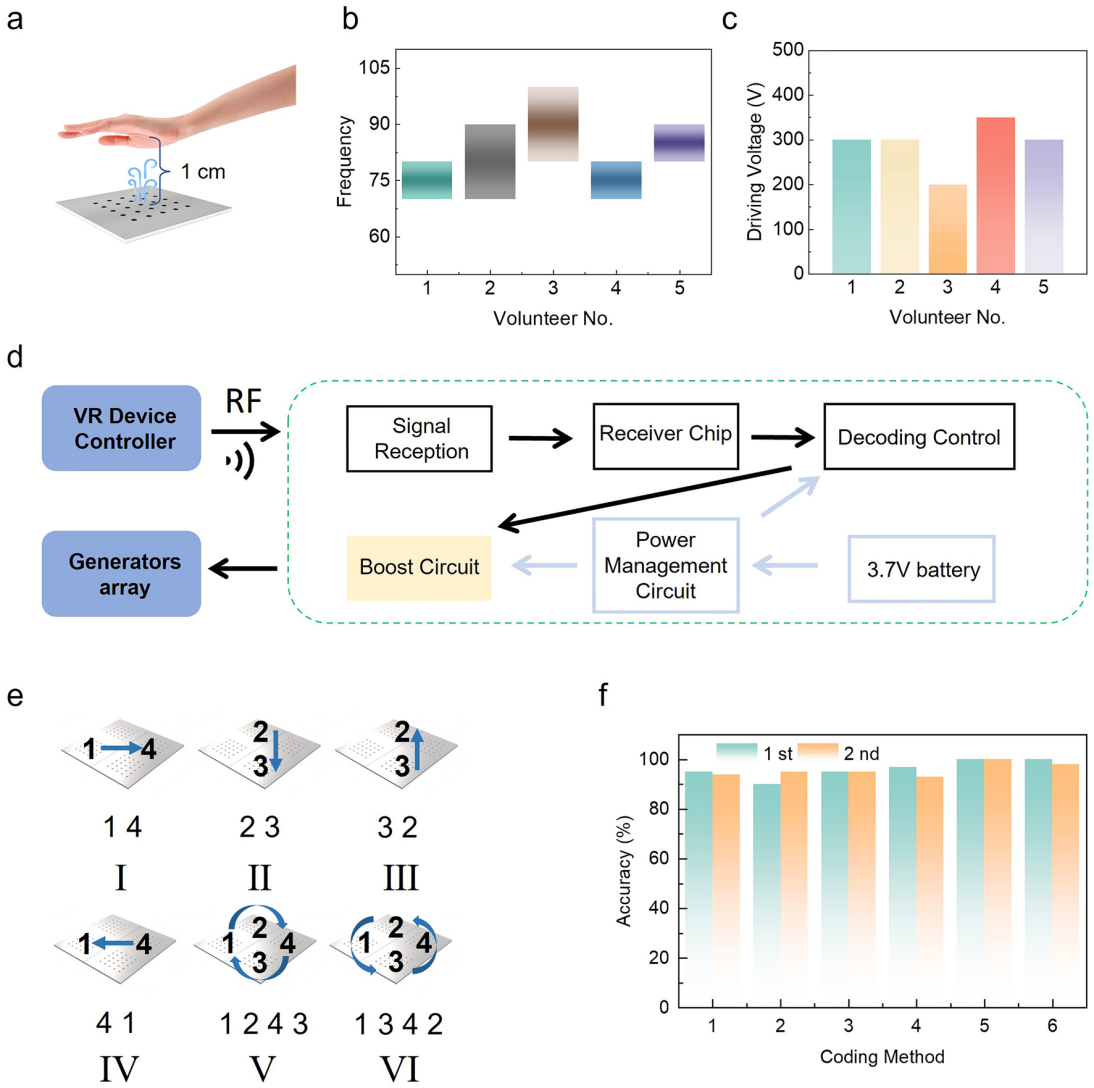
Non-contact breeze-sense feedback for coding information transfer. **a** Schematic diagram for the volunteer’s test. **b** Optimal frequency range for six volunteers measured at fixed *V*_*P*–*P*_ of 1000 V. **c** Threshold driving voltage that could be felt by 6 volunteers, under optimal frequency range of 70–90 Hz. **d** Flowchart of the control system for the coding breeze-sense information transfer demonstration. **e** Schematic of the six coding methods, in which a 2 × 2 generators array with each unit size of 2 × 2 cm^2^ is used. **f** Accuracy of identifying 6 encodings by two volunteers, when V_P-P_ and frequency are 500 V and ~ 160 Hz

To validate the effect of breeze feedback on human skin receptors, a schematic of a 6 × 6 cm^2^ breeze-sense generator with the position of hand placement (1 cm) we set in the volunteer test is illustrated in Fig. 4a. The size of the generator is designed to cover the most area of a human palm. During the test, five volunteers are asked to feel air flow pressure when varying the driving frequency (1–300 Hz) at a fixed driving voltage (*V*_*P*–*P*_ = 1000 V) and figure out the range of frequency which they can feel the strongest feedback sense. Most of the volunteers feel maximum air flow pressure at frequencies ranging from 70 to 90 Hz, as shown in Fig. 4b. These results align with the resonant frequency of the 6 × 6 cm^2^ breeze-sense generator, as shown in Fig. S12c. Moreover, under the most sensitive frequency range, we test the threshold driving voltage that could be felt by volunteers. As shown in Fig. 4c, the lowest driving voltage that could be felt by the 5 volunteers is in the range of 200–350 V.

To further verify the ability of the breeze-sense generators array to transfer coding information, we design a compact system to control the generators array. Figure 4d illustrates the flowchart of the control system. In specific, the RF signal transmitted by the controller contains the information from VR environments or manual control; then, the receiver module decodes the reception and sends it to the booster circuit to activate the breeze-sense generators. The whole system is portable and powered by a 3.7 V battery. The optical image of the control system is shown in Fig. S17, and the details of the design are shown in Fig S18. To protect the system, we also design a flexible case (Fig. S19). The endurance of the system is up to 4 h with a small-sized 900 mAh Li-battery, which is enough for normal usage (Fig. S20). Here, we use a 2 × 2 generators array to demonstrate the six encoding modes for information transfer, in which each generator is 2 × 2 cm^2^ and the thickness is ~ 950 µm (Fig. S21a). The distance between each generator is 4 mm. The flexibility of the array is also excellent (Fig. S21b). The six coding methods are visualized in Fig. 4e, which are coded by the sequential working of specific generators (model I: 1 → 4; model II: 2 → 3; model III: 3 → 2; model IV: 4 → 1; model V: 1 → 2 → 4 → 3; model VI: 1 → 3 → 4 → 2). Two volunteers are asked to identify the six encodings (Movie S2), and the overall accuracy is than 96% when *V*_*P*–*P*_ is 500 V and frequency is ~ 160 Hz, as shown in Fig. 4f. On the other hand, we ask the volunteers to identify two nearby generators working at the same time (model I: 1&4; model II: 2&3; model III: 1&3; model IV: 2&4), and the accuracy is up to ~ 98% (Fig. S22 and Movie S3). Above results indicate the low interference of our breeze-sense generators array and potential application of coding information transfer.

### Non-Contact Breeze-Sense Feedback Demonstrations in VR

**Fig. 5:**
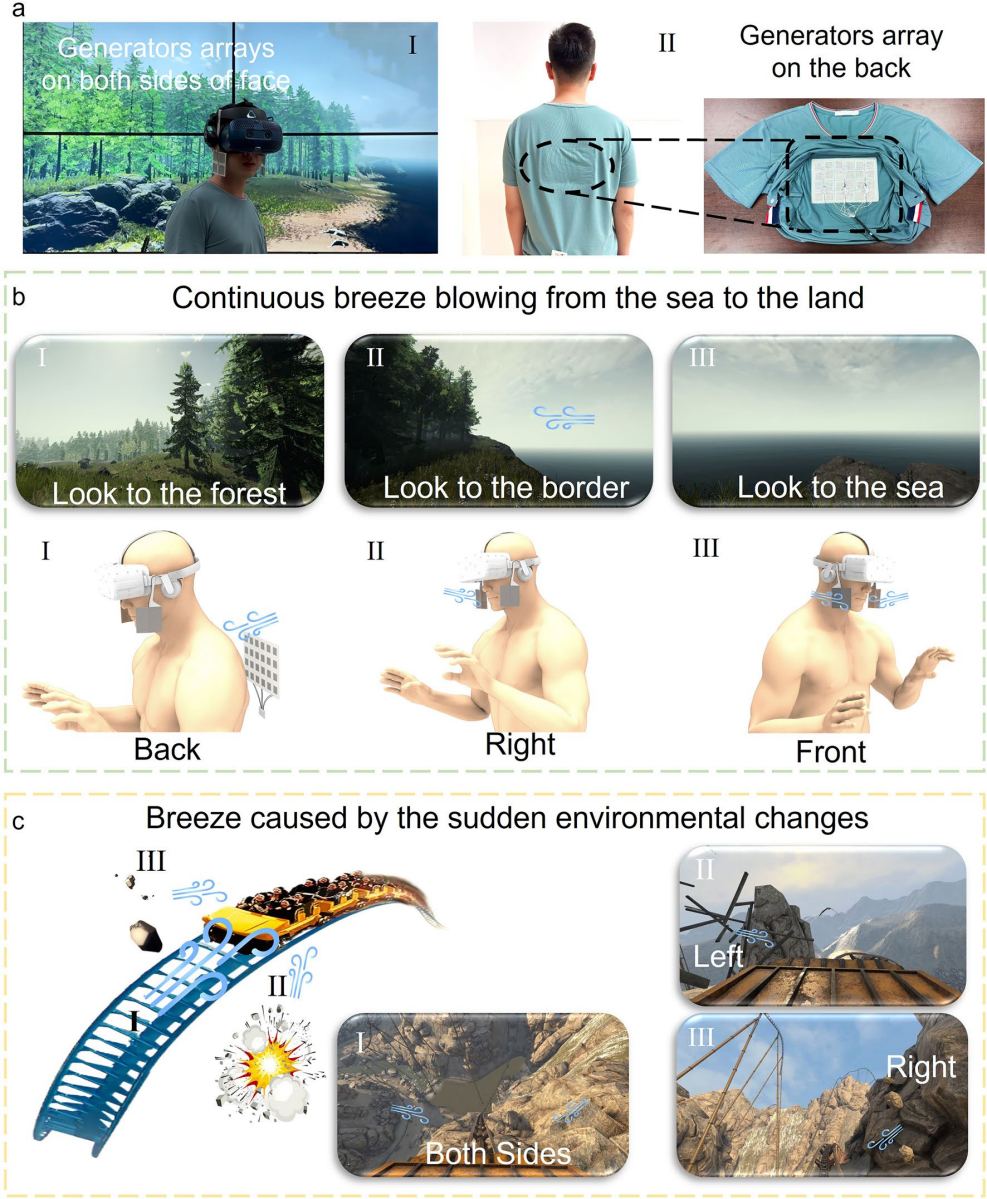
Non-contact breeze-sense feedback demonstrations in VR. **a** Image illustrating a non-contact breeze-sense feedback wearable system integrated with VR devices, I: two 2 × 2 generators arrays locate on both sides of the volunteer’s face; II: a 4 × 6 generators array locates on the back. **b** Volunteer feels the continuous breeze blowing from the sea to the land. **c** Volunteer feels the breeze caused by the sudden environmental changes

To demonstrate the wearability in combination with VR, a non-contact breeze-sense feedback wearable system is integrated with VR devices worn by a volunteer, as shown in Fig. 5a. Two 2 × 2 breeze-sense generators arrays locate at the left and right sides of the volunteer’s face (Fig. 5a-I), and the working area of each unit is 3 × 3 cm^2^ (Fig. S23). These devices help the volunteers to distinguish the breeze from the left or right. Another 4 × 6 breeze-sense generators array (the working area of each unit is 2 × 2 cm^2^, Fig. S24) locates on the back of the volunteer (Fig. 5a-II). This device is designed to give volunteers a breeze-sense on the back. We use above three arrays to make the volunteer perceive the continuous or sudden breeze senses from various orientations in VR environments. When arrays are activated, the driving voltage and frequency are 500 *V*_*P*–*P*_ and 160 Hz, respectively. It should be noted that VR devices are normally used in an indoor environment. The airflow in indoor environment is generally random or continuous, making it easy to be distinguished from the coding airflow generated by our device. Therefore, the interference caused by the surrounding airflow is very limited.

In the first demonstration, the volunteer’s virtual location is set at a place where the forest meets the sea. The virtual environment involves continuous breeze blowing from the sea to the land. The three breeze orientations we set up in VR environment are shown in Fig. 5b and Movie S4. I. When the volunteer looks at the forest, the breeze is blowing from the back, and the array at the back is activated; II. When the volunteer looks to the border, the sea is in the right side of the volunteer, and the array on the right side of the volunteer's face is activated; III. When the volunteer looks to the sea, the two arrays on the left and right sides of the volunteer's face are simultaneously activated. Another scenario of riding a roller coaster in the VR environment is shown in Fig. 5c and, Movie S5 in which the volunteer can feel the breeze caused by the sudden environmental changes. I. The array locate on both sides of the volunteer’s face are activate when the roller coaster dives down; II. When there is an explosion on the left, the array locates on the left side of the volunteer’s face is activated; III. The array locates on the right side of the volunteer’s face activates when there is a rock falling on the right side.

## Conclusions

In summary, thin and flexible breeze-sense generators are proposed to make the users perceive the non-contact haptic feedback in the VR environments. The air flow pressure output performances are improved by well designing the key parameters. As a result, the breeze-sense generators generate significant air flow pressure output of ~ 163 Pa that can easily be sensed by human skin and have an overall thickness of less than 2 mm and good flexibility to maintain more than 50% of the initial output value when bent at 50°. These features make our devices be appropriate for integrating with existing AR/VR systems. During continuous 5 working hours, the air flow pressure outputs have less than 10% variation, proving the excellent durability which is essential for practicability. In volunteers’ test, the breeze-sense generators array is successfully demonstrated to transfer encoding information. Moreover, a non-contact breeze-sense feedback wearable system is developed to provide continuous or sudden breeze senses from various orientations for the volunteer in VR environments. Further research efforts will focus on the following points: (1) reducing the driving voltage and enhancing the robustness of operation in harsh environments to further increase the overall practicality and (2) developing the AR/VR scenarios that can deliver encoding breeze-sense with good resolution, such as a mother blows across the hand of a baby.

## Supplementary Information

Below is the link to the electronic supplementary material.

Supplementary file1 (MP4 1185 KB)

Supplementary file2 (MP4 3343 KB)

Supplementary file3 (MP4 3498 KB)

Supplementary file4 (MP4 7541 KB)

Supplementary file5 (DOCX 9663 KB)

Supplementary file6 (MP4 10689 KB)
