# Supplementary material for: Thin and Flexible Breeze-Sense Generators for Non-Contact Haptic Feedback in Virtual Reality
Source: Nanomicro Lett. 2025 Feb 13;17:144. doi: 10.1007/s40820-025-01670-y (PMC11825423; doi:10.1007/s40820-025-01670-y)
Supplement: Supplementary file 5 — Supplementary file5 (DOCX 9663 KB) [file 40820_2025_1670_MOESM5_ESM.docx]

Supporting Information for

**Thin and Flexible Breeze-Sense Generators for Non-Contact Haptic Feedback in Virtual Reality**

Kaijun Zhang^1^, Zhe Liu^1^, Yexi Zhou^1^, Zhaoyang Li^1^, Dazhe Zhao^1^, Xiao Guan^1^, Tianjun Lan^1^, Yanting Gong^1^, Bingpu Zhou^2^, Junwen Zhong^1^*

^1^ Department of Electromechanical Engineering and Centre for Artificial Intelligence and Robotics, University of Macau, Macau SAR, 999078, P. R. China

^2^ Joint Key Laboratory of the Ministry of Education, Institute of Applied Physics and Materials Engineering, University of Macau, Macau SAR, 999078, P. R. China

*Corresponding author. E-mail: [junwenzhong@um.edu.mo](mailto:junwenzhong@um.edu.mo) (Junwen Zhong)

**Supplementary Figures and Table**


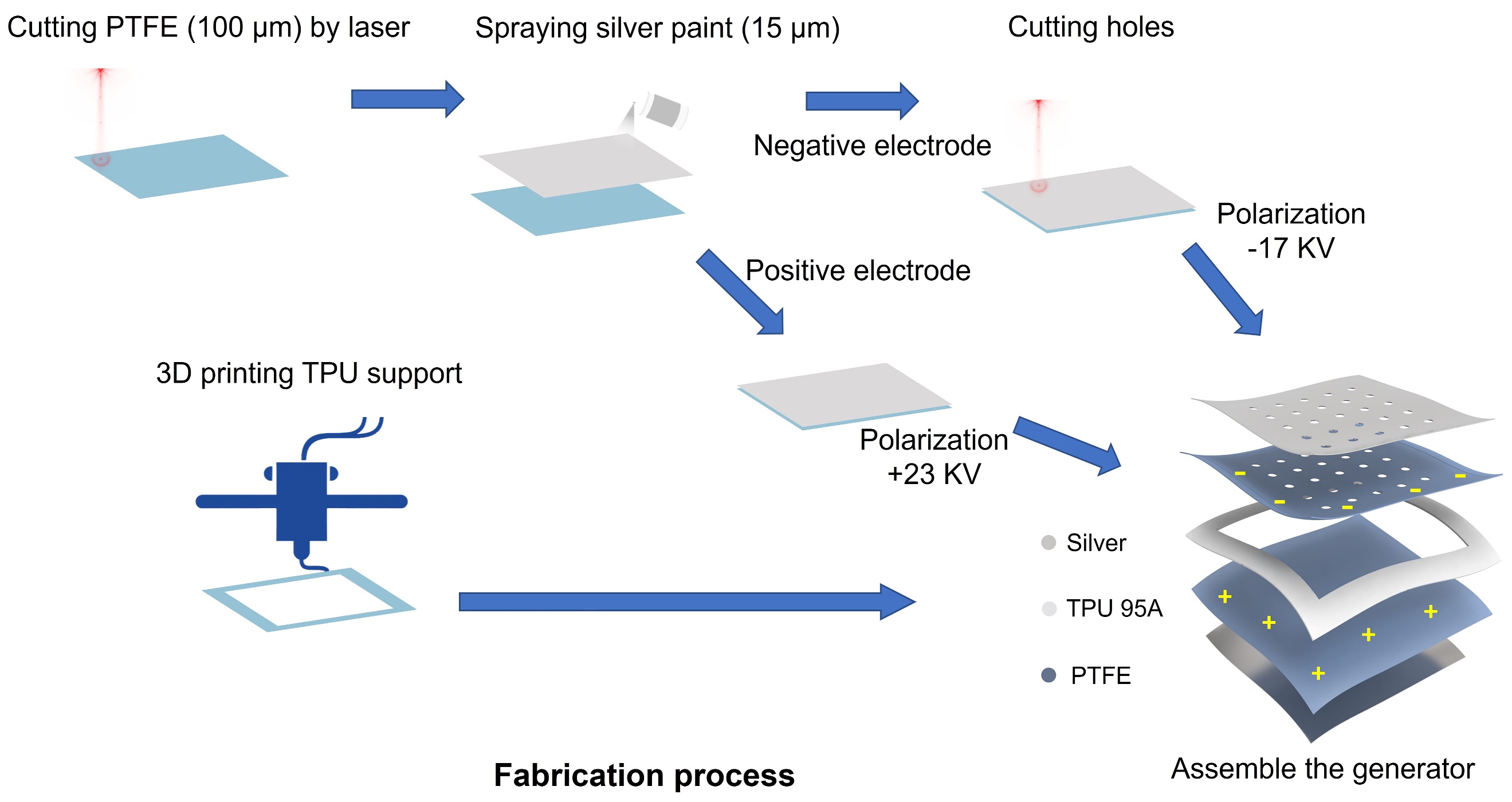


**Fig. S1** The detail fabrication processes of a breeze-sense generator


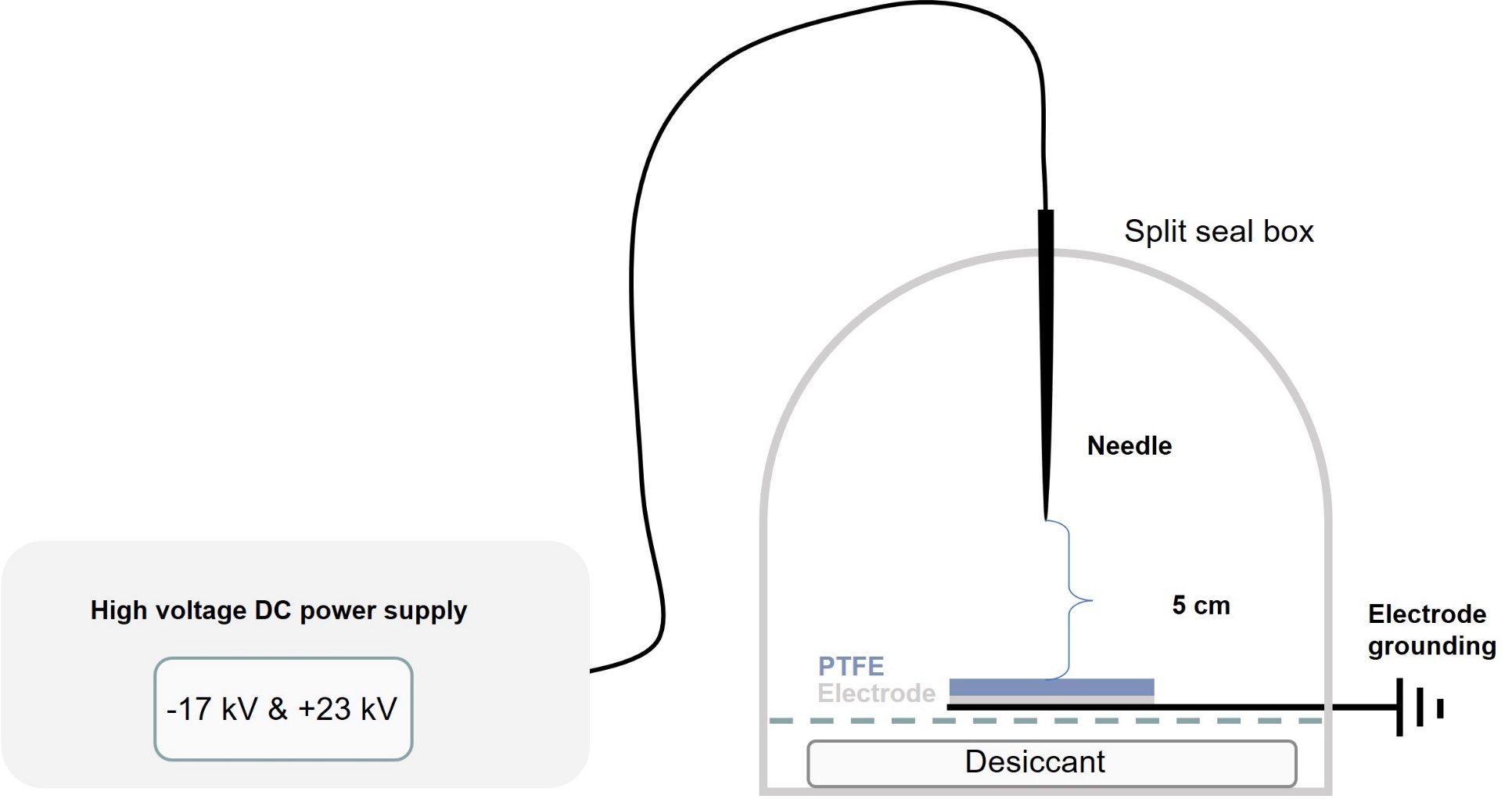


**Fig. S2** Schematic diagram of Corona charging setup


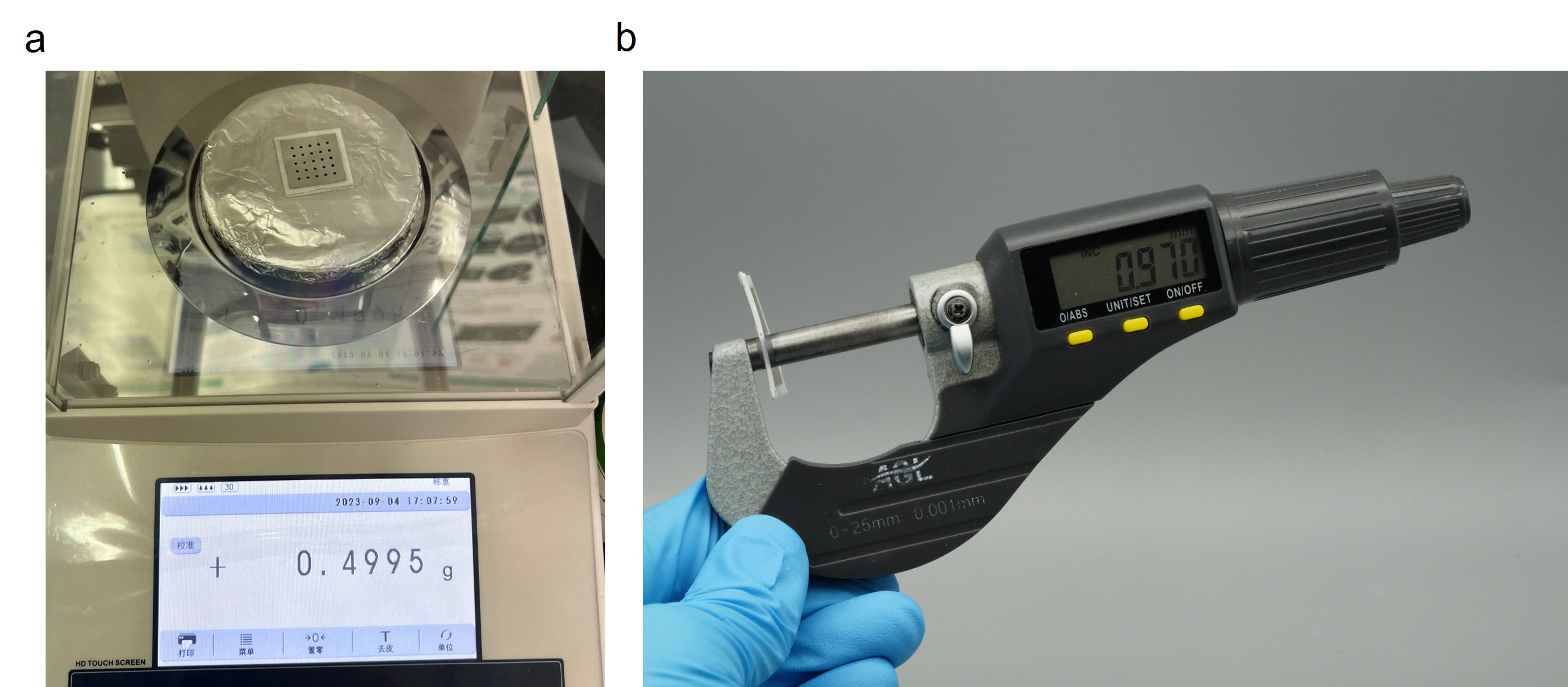


**Fig. S3 a** Image of a 2×2 cm^2^ breeze-sense generator with a weight of ~ 0.4495g. **b** Image of a 2×2 cm^2^ breeze-sense generator with a thickness of ~ 970 µm


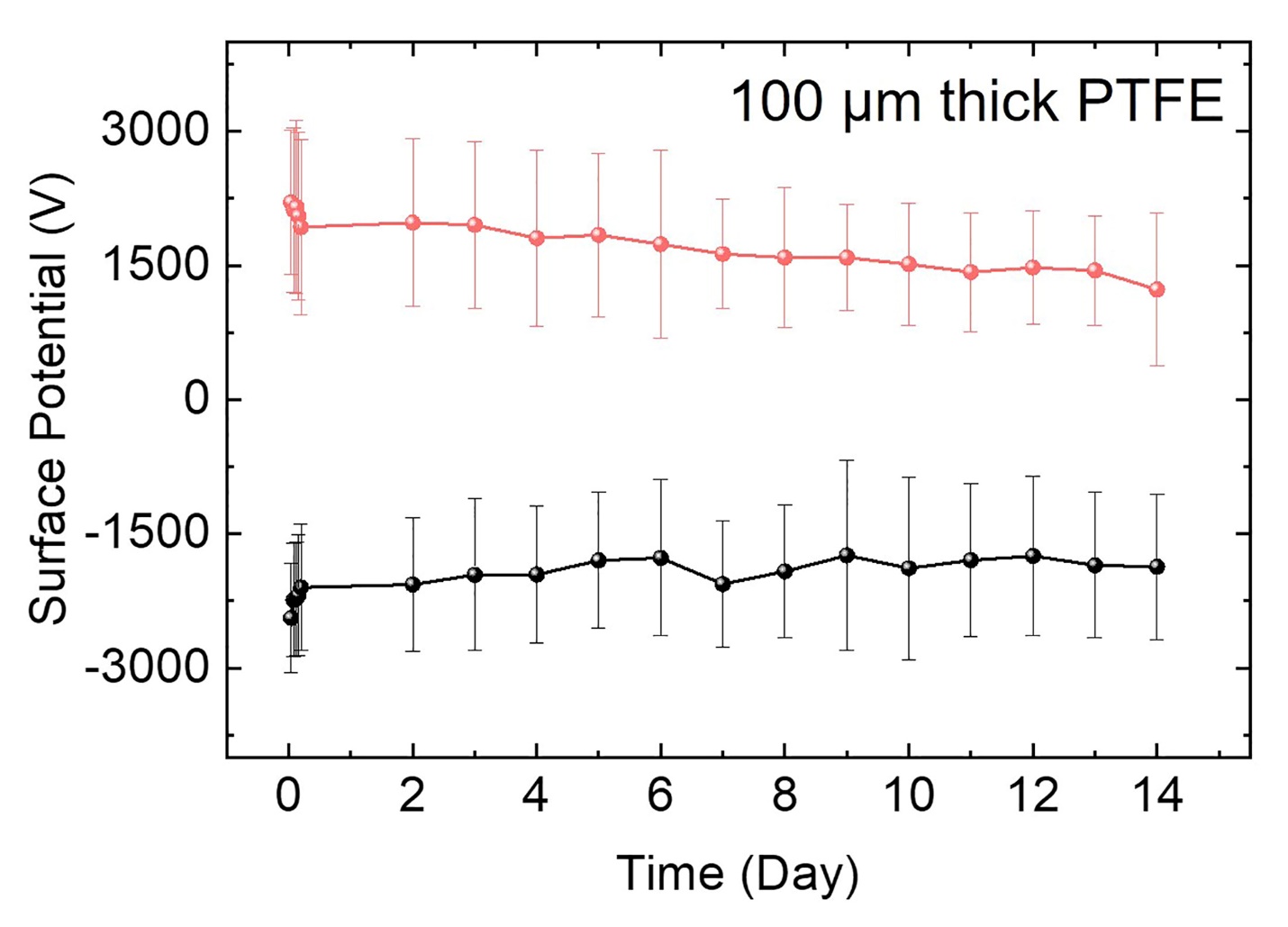


**Fig. S4** Surface potential values *versus* time curves for positive and negative Corona charged PTFE/Ag films, and the testing time is lasted for 14 days


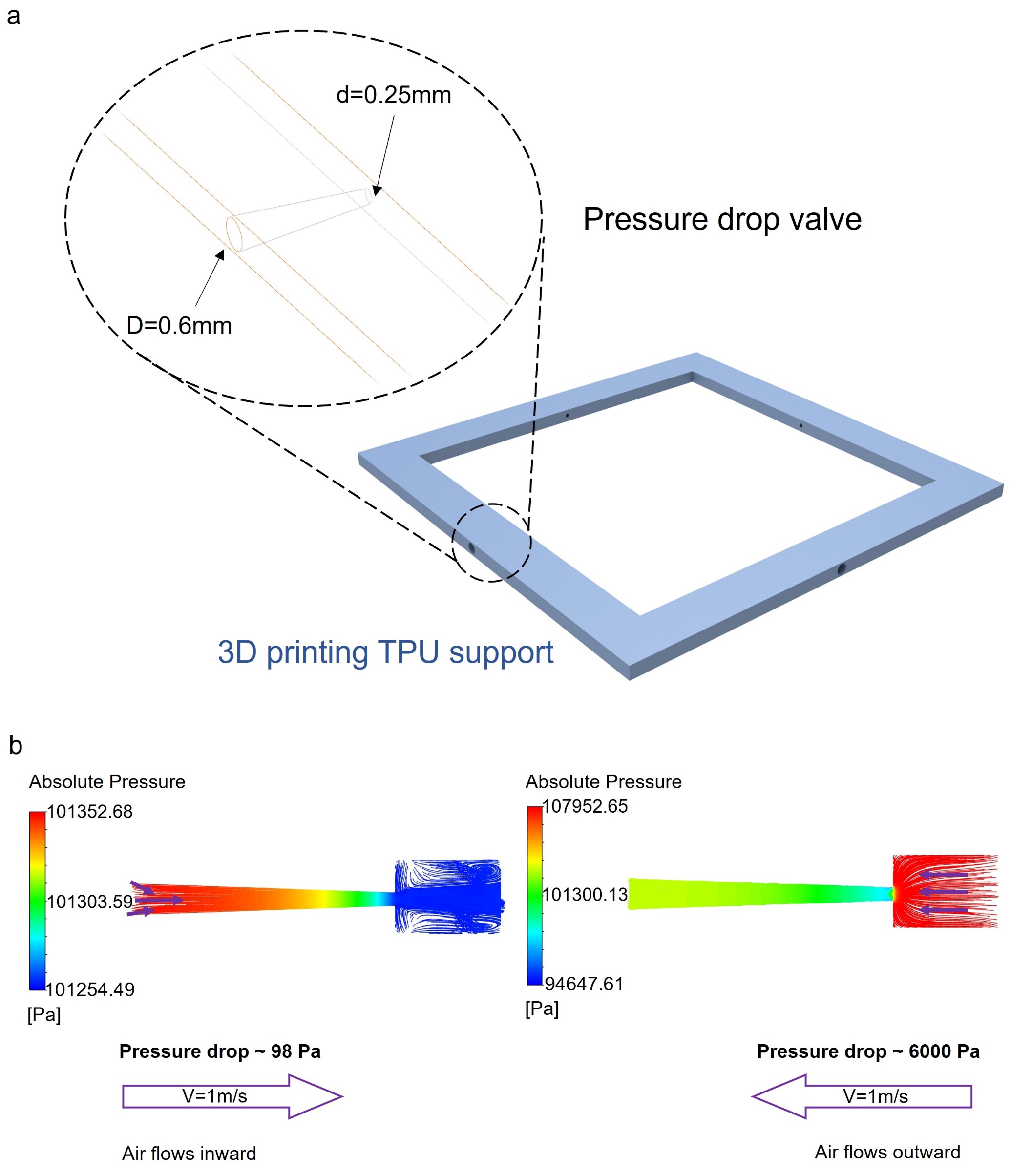


**Fig. S5 a** Schematic diagram and **b** fluid simulation result of pressure drop valve


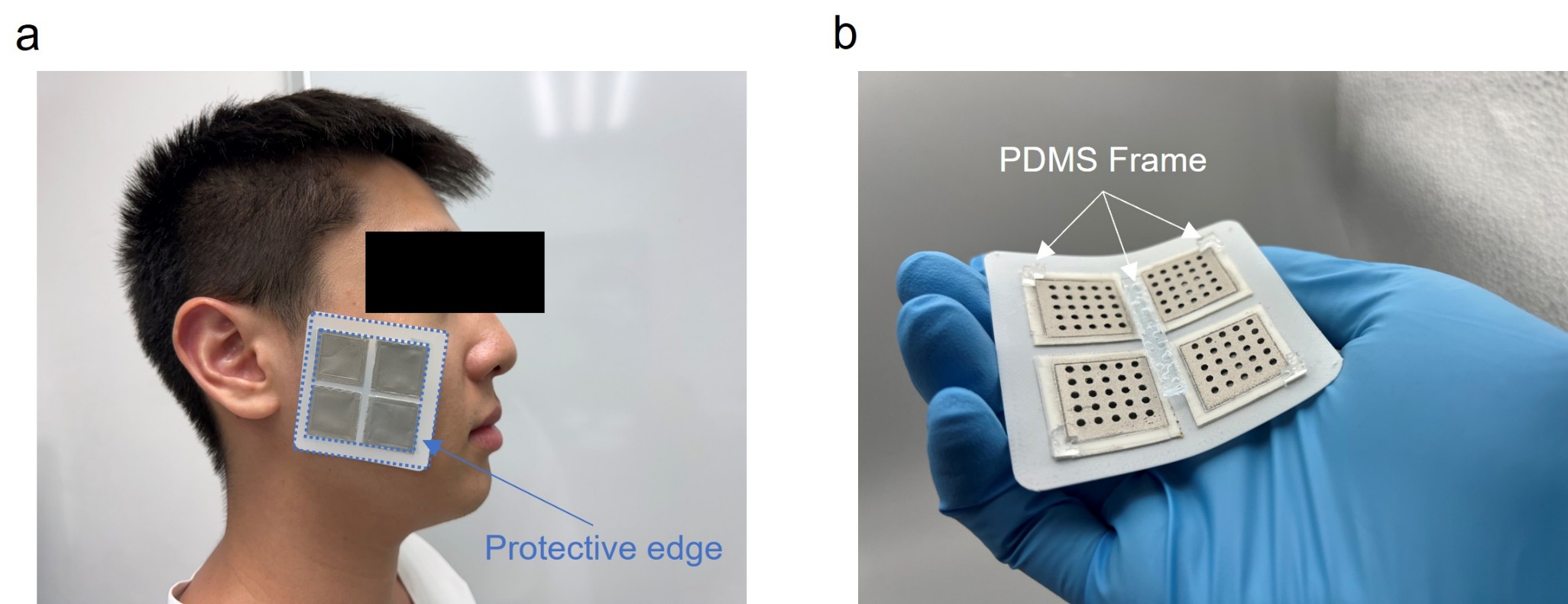


**Fig. S6 a** Optical image of 2×2 generators array when in wearable conditions and **b** PDMS frame position for the array


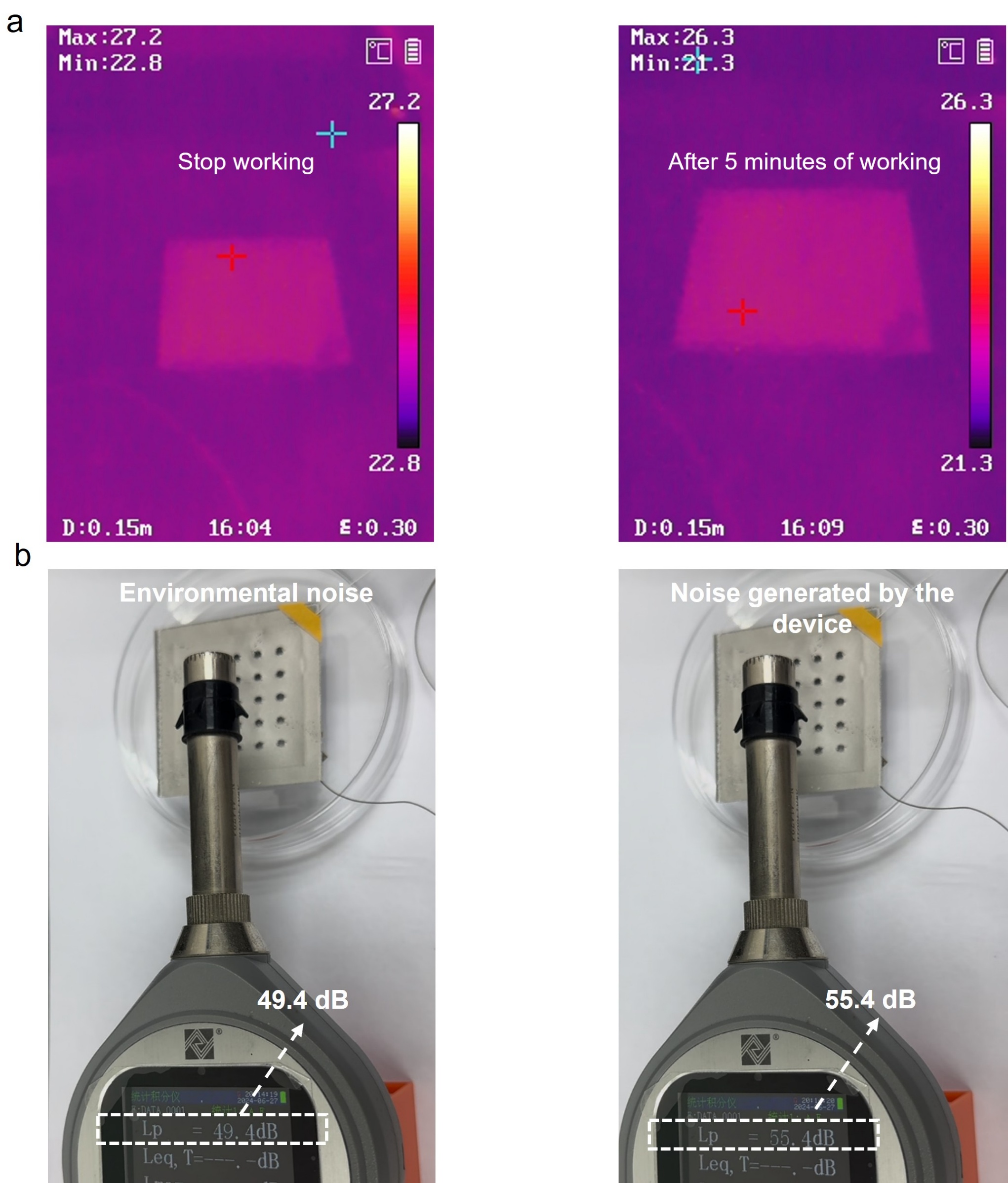


**Fig. S7** Images of **a** thermal and acoustic **b** characteristics of a working breeze-sense generator, at a frequency of 160 Hz and V_P-P_ of 500 V


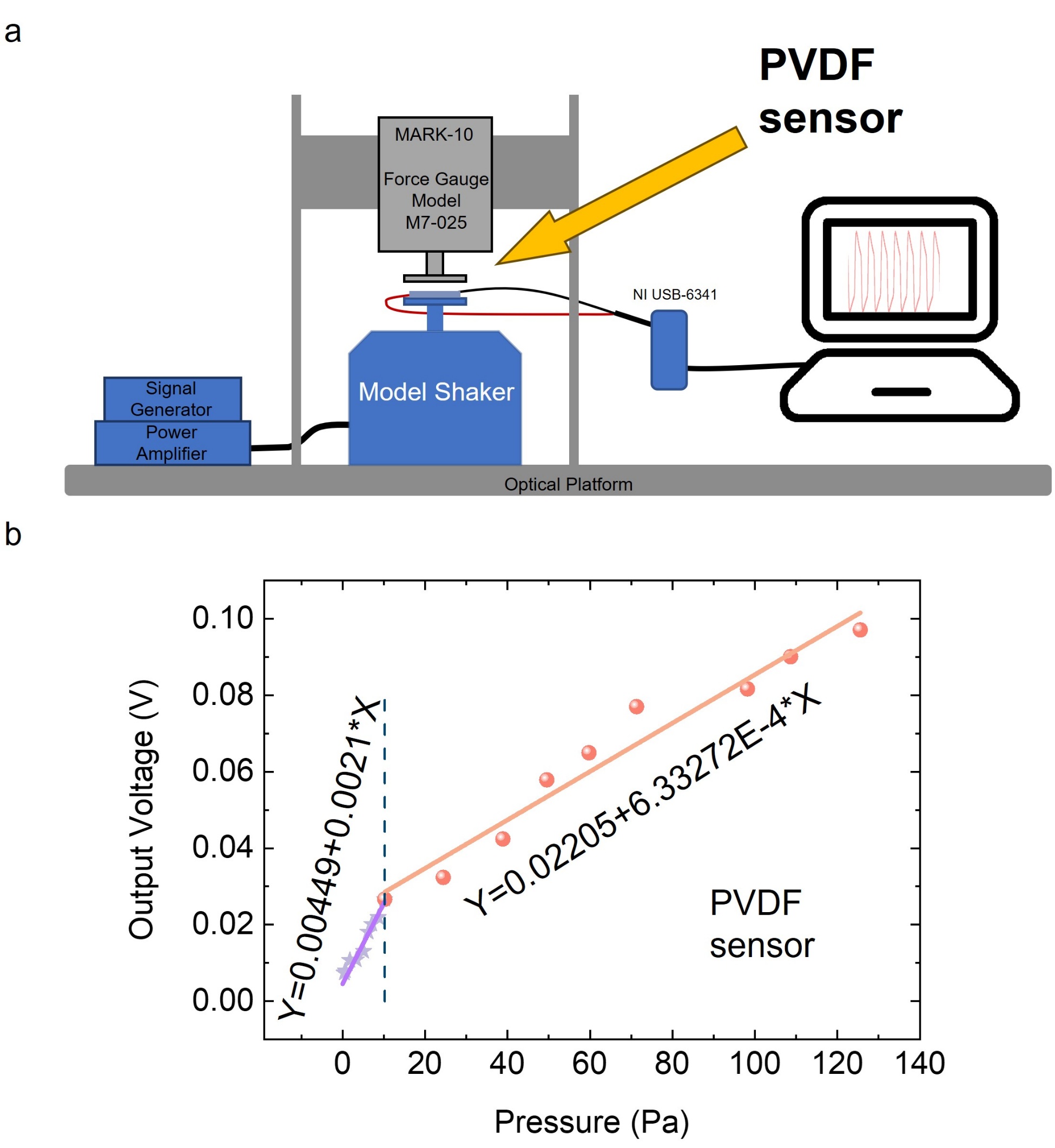


**Fig. S8** PVDF pressure sensor calibration. **a** The schematic of measuring the output voltage of PVDF sensor by a model shaker. **b** The output voltage curves of PVDF pressure sensor under different pressures


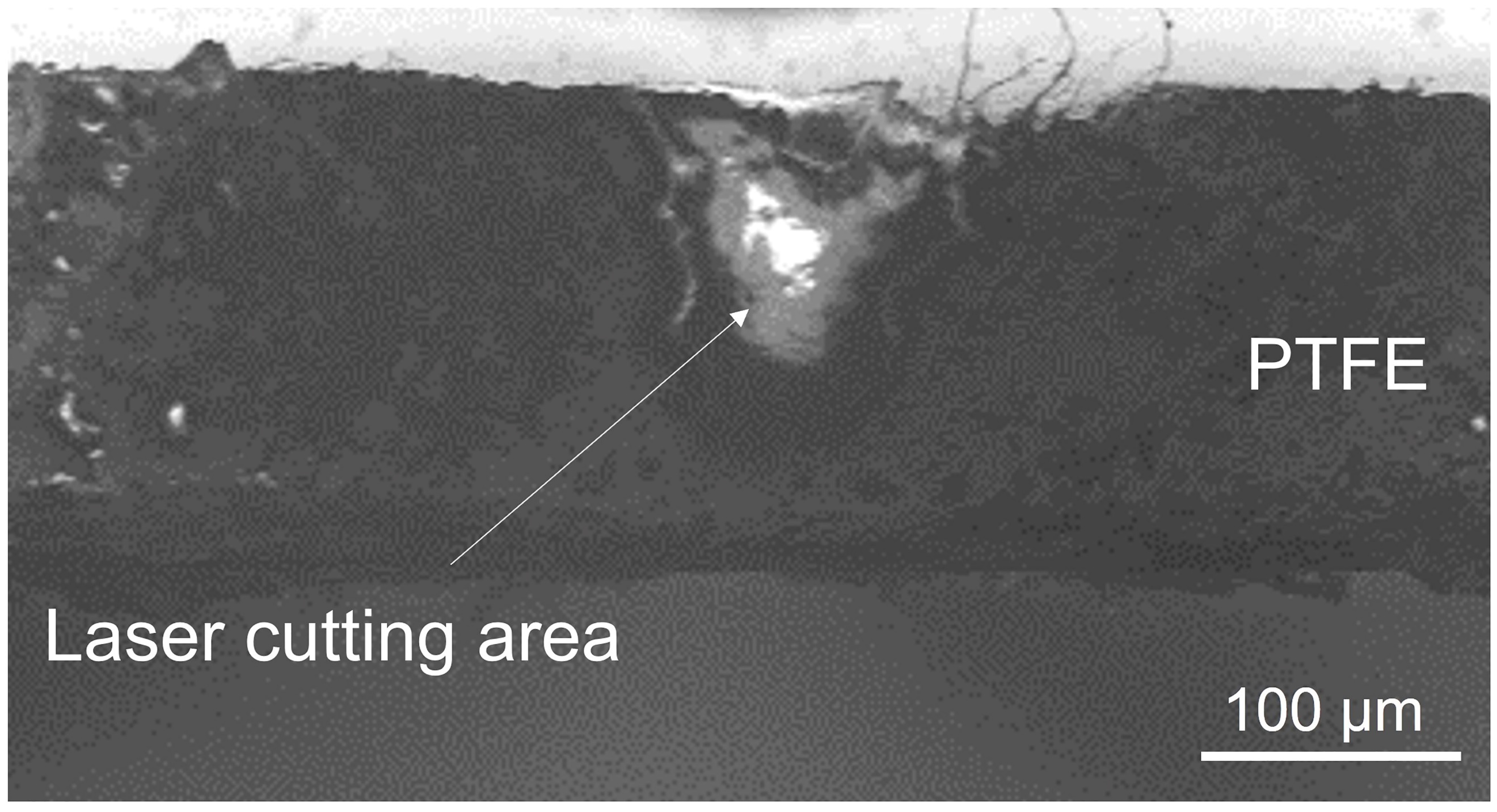


**Fig. S9** The cross-section SEM image of the PTFE films with laser cutting boundary


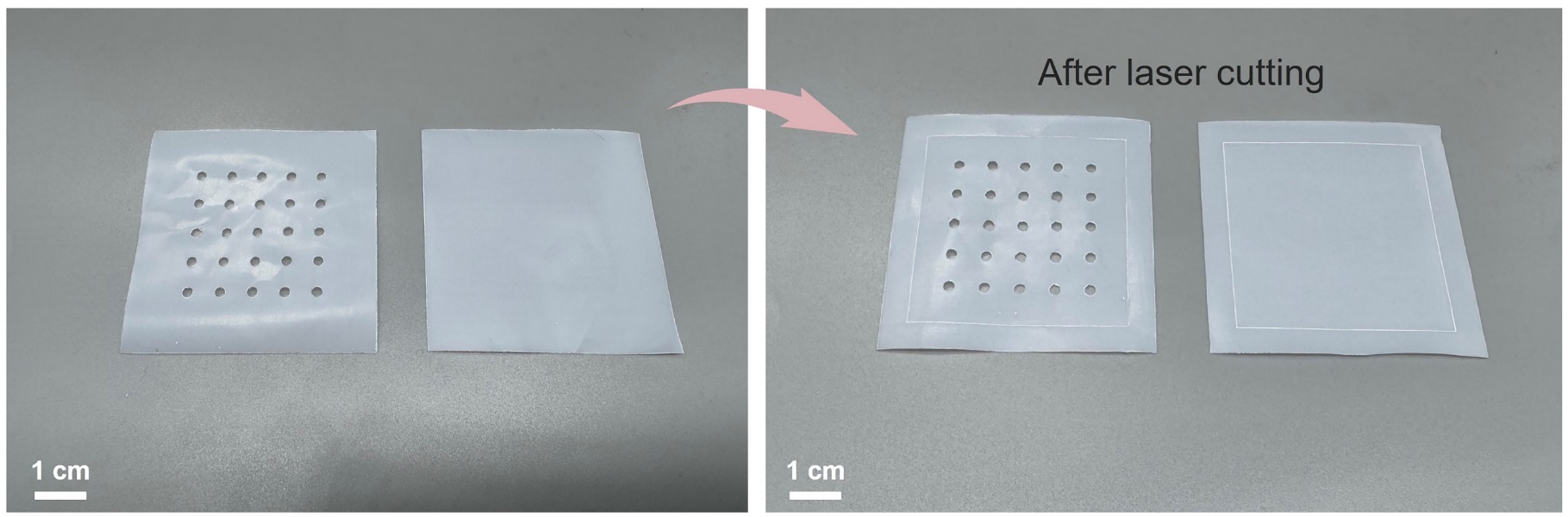


**Fig. S10** The images of PTFE films before and after laser cutting


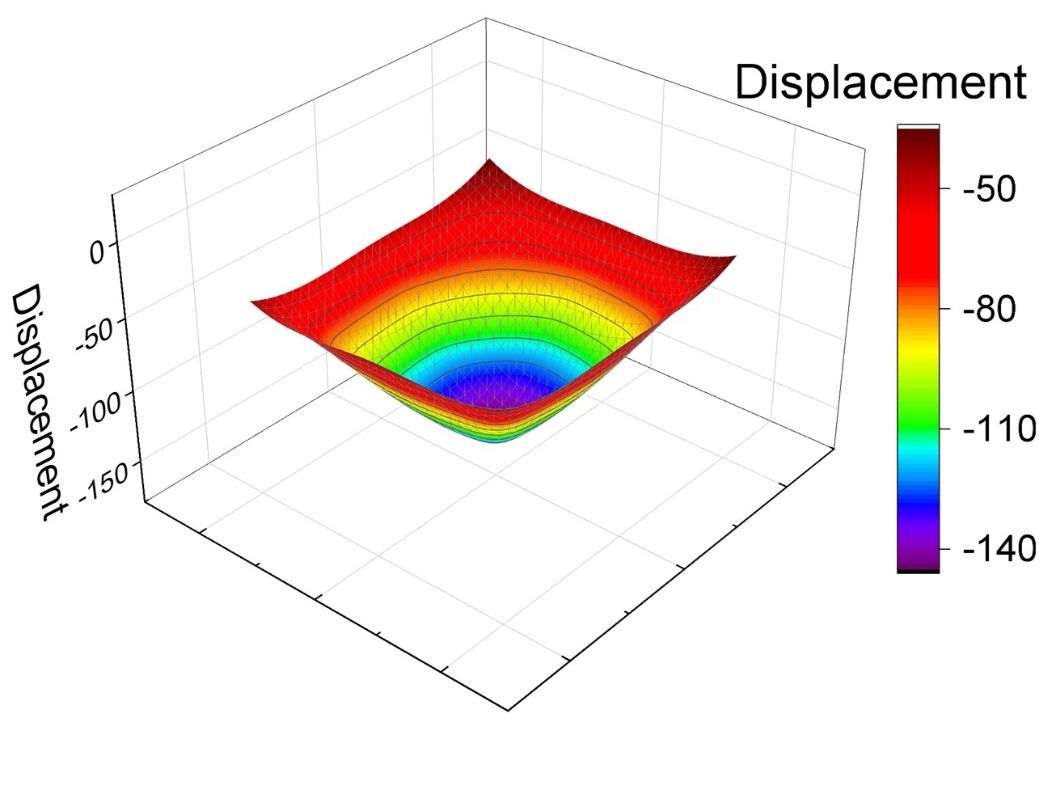


**Fig. S11** Vibration displacement distribution on the lower layer of a 4*4 cm^2^ breeze-sense generator (V_P-P_ = 500 V, frequency = 160 Hz)


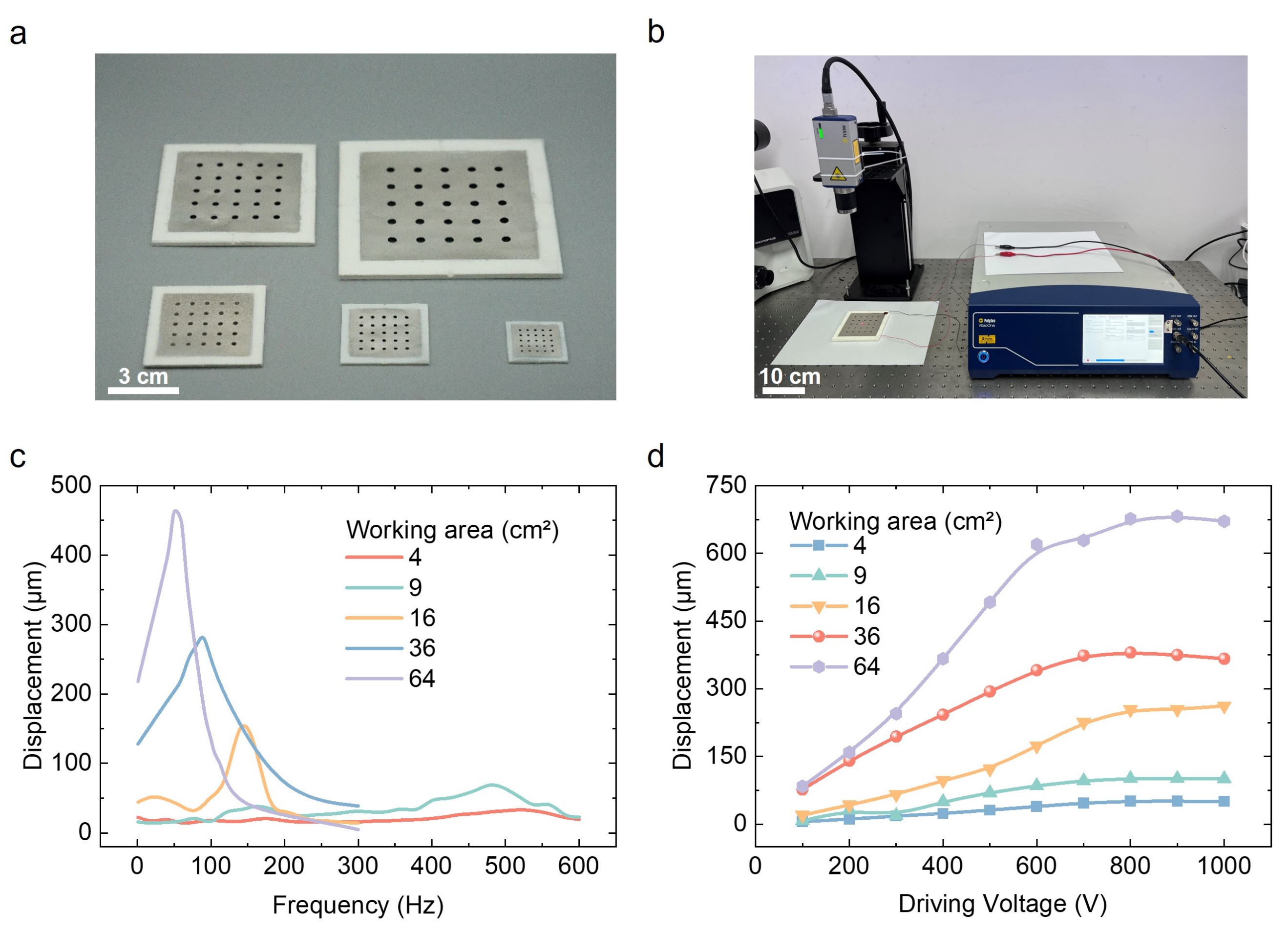


**Fig. S12** **a** Image of breeze-sense generators with different working areas. **b** The image shows the displacement testing setup. **C** The top PFTE/Ag layer displacement of breeze-sense generator with different working areas when driving frequency changes from 1-600 Hz (V_P-P_ = 500 V). **d** The top PFTE/Ag layer displacement of breeze-sense generators with different working areas when changing the V_P-P_ at the resonant frequency


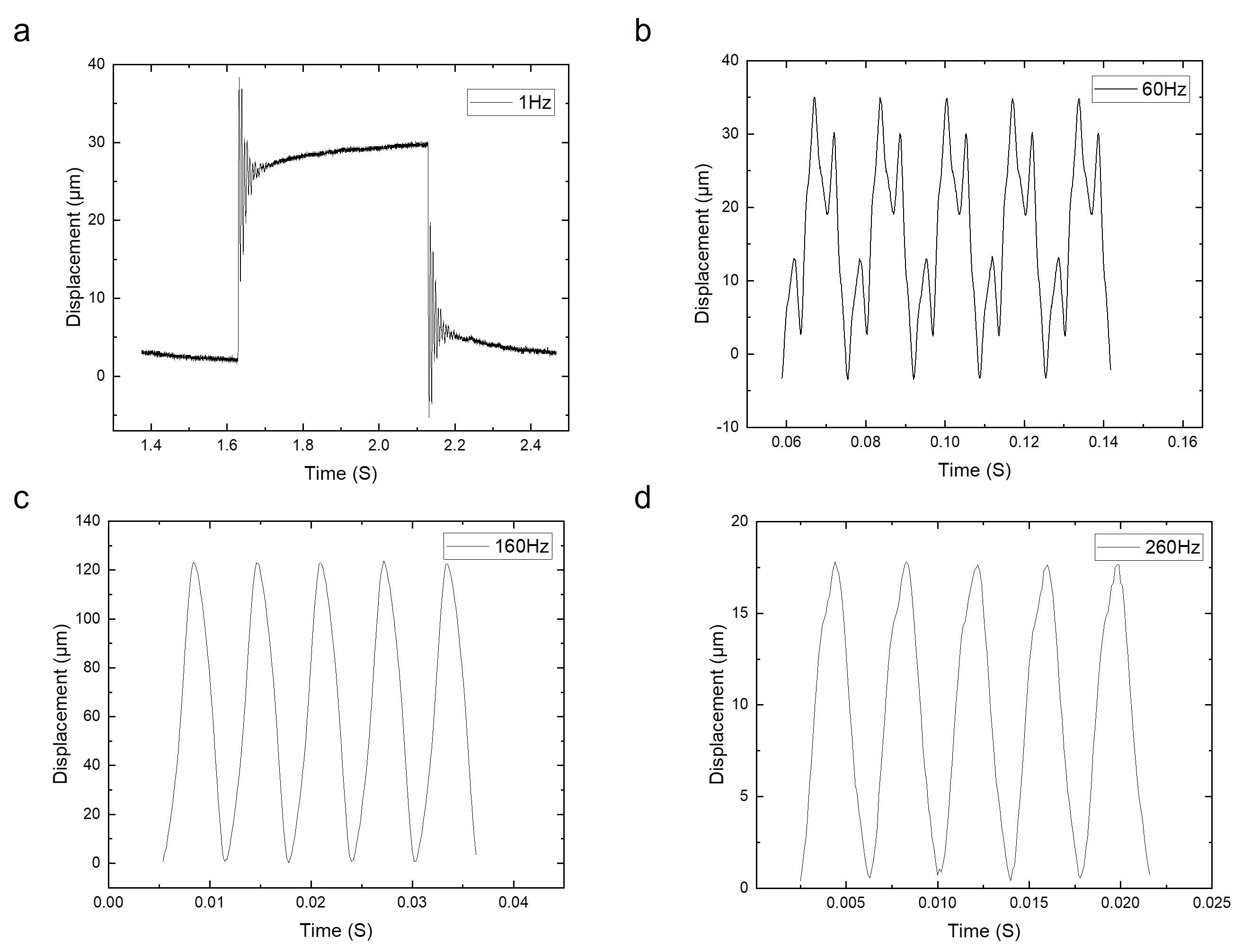


**Fig. S13** The displacement curves of top PTFE/Ag layer driven by 4 different frequencies (V_P-P_ = 500 V). The driving frequencies are **a** 1 Hz, **b** 60 Hz, **c**160 Hz, and **d** 200 Hz


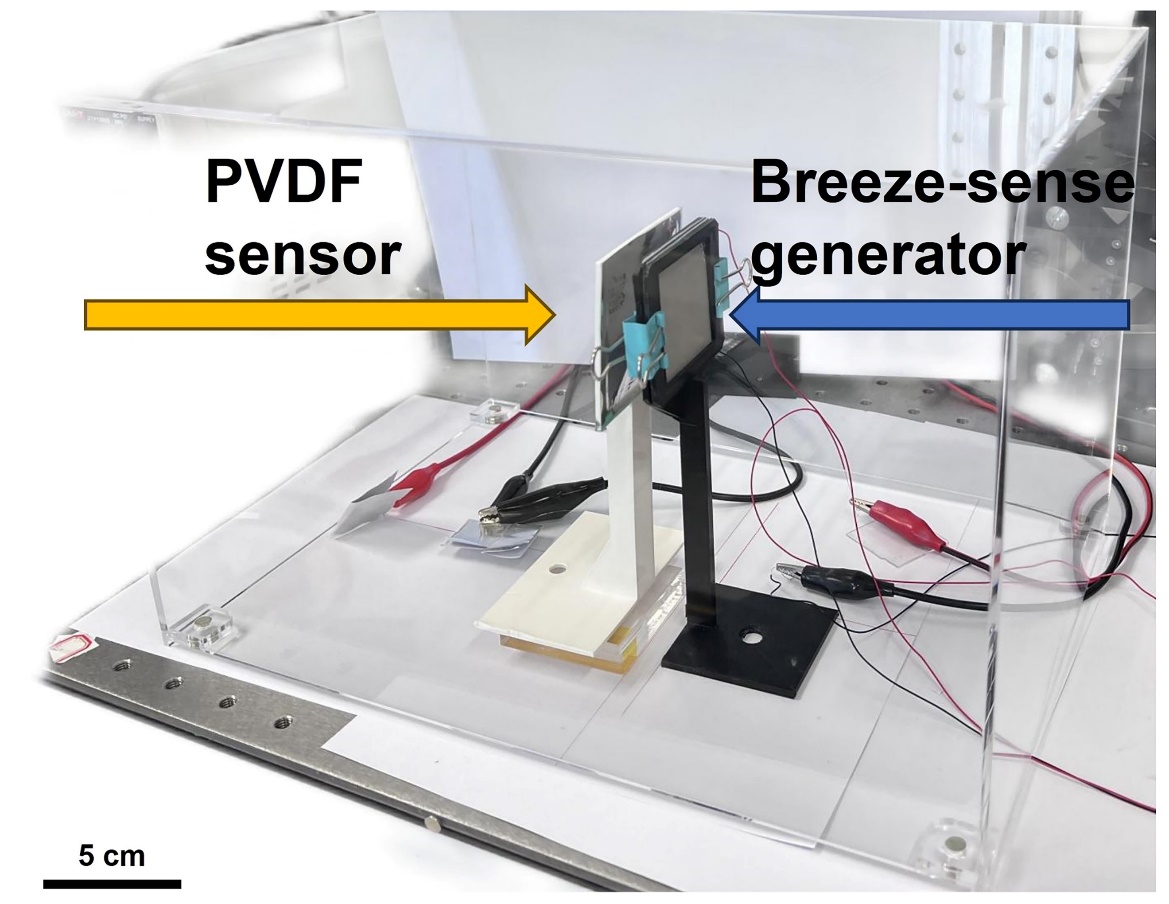


**Fig. S14** The image of the breeze-sense generator output testing setup


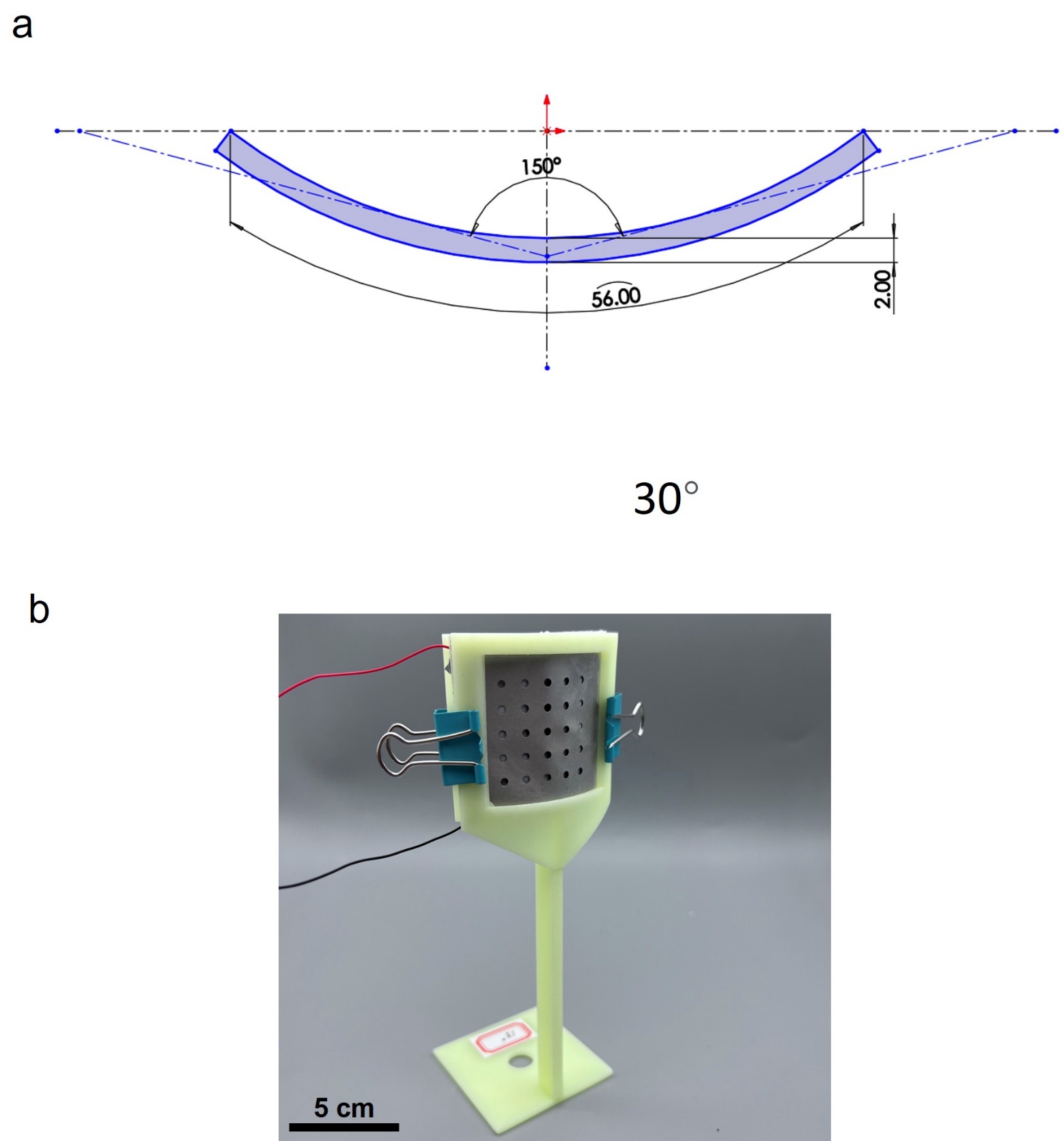


**Fig. S15** Schematic diagram of **a** 30-degree bent definition and **b** image of a 3D printed bracket for holding a breeze-sense generator


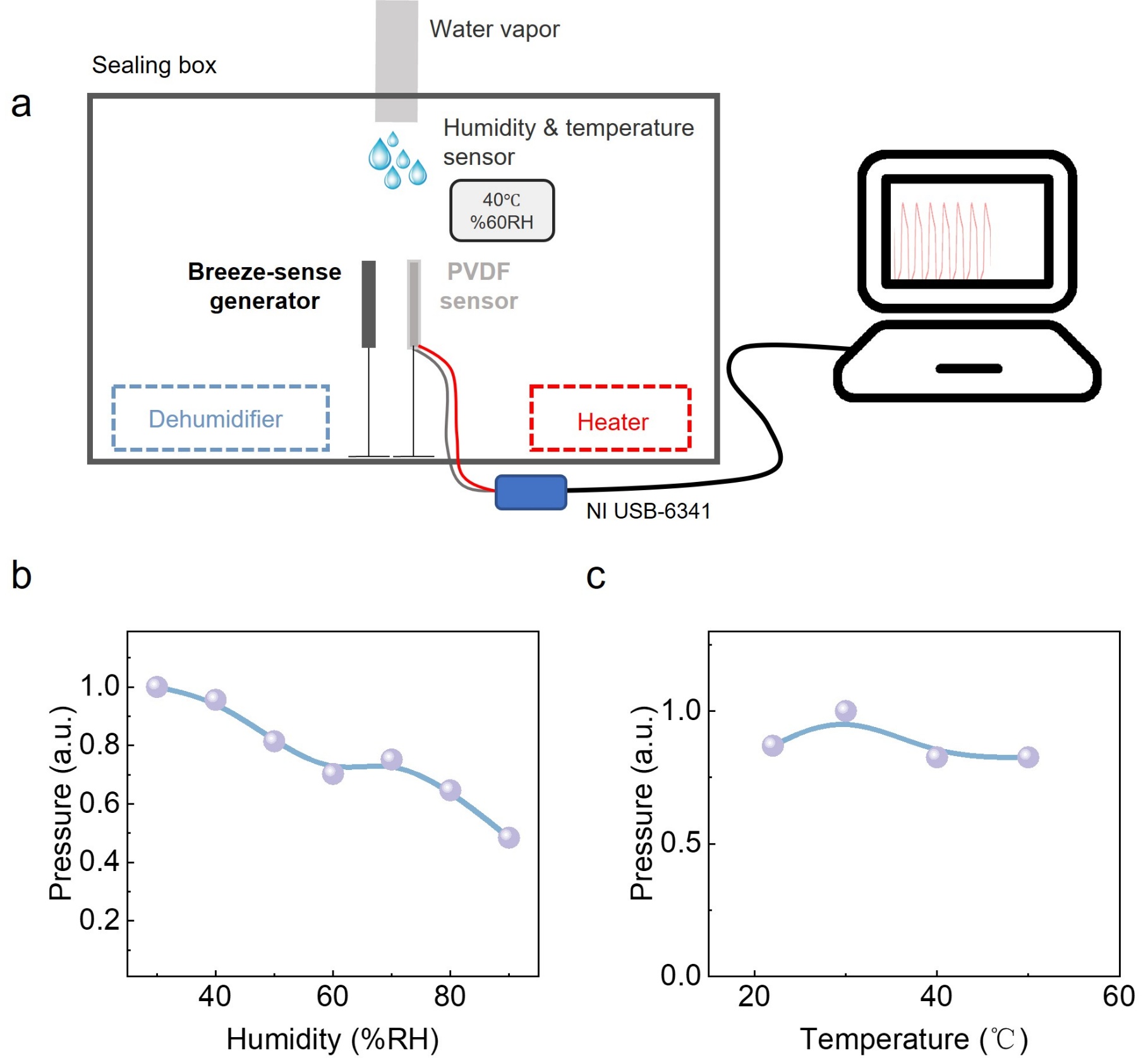


**Fig. S16 a** Schematic illustration of humidity and temperature testing. The device is placed inside a sealed box which has a dehumidifier inside to adjust the humidity, and a hygrometer is used for monitoring humidity. There are also humidifiers and heating platforms to control the temperature and humidity. **b** Influence of humidity to output pressure (from 30 to 90%RH). **c** Influence of temperature to output pressure (from 20 to 50 ℃)


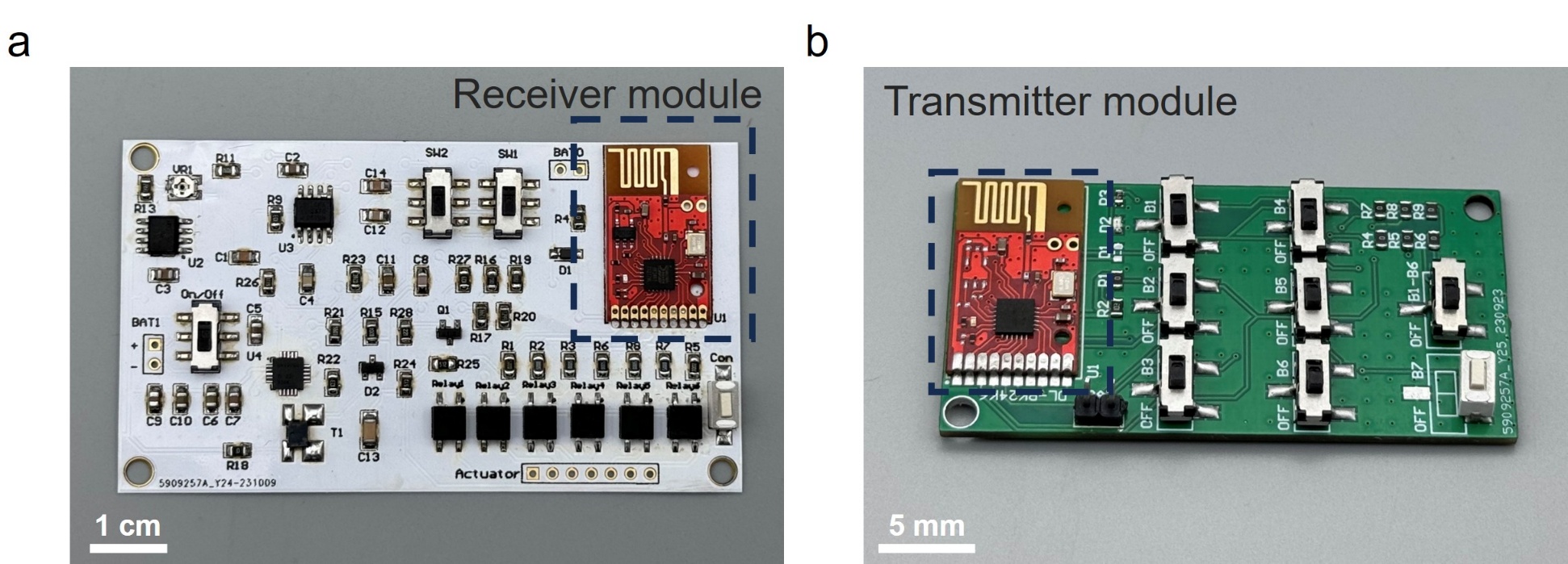


**Fig. S17** The images of **a** the decoding and boost circuit and **b** the coding circuit
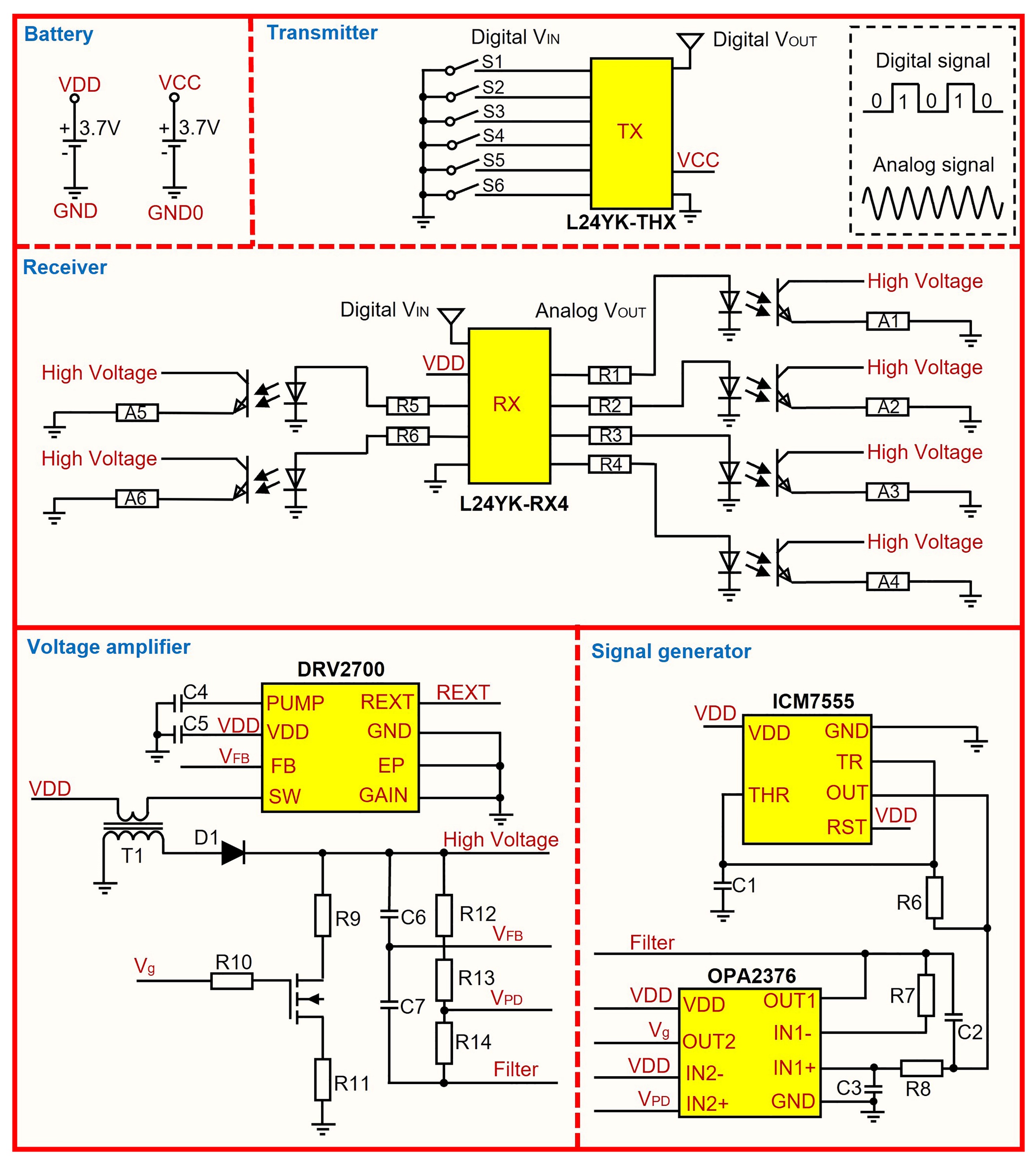


**Fig. S18** Circuit diagram of boost circuit and coding circuit


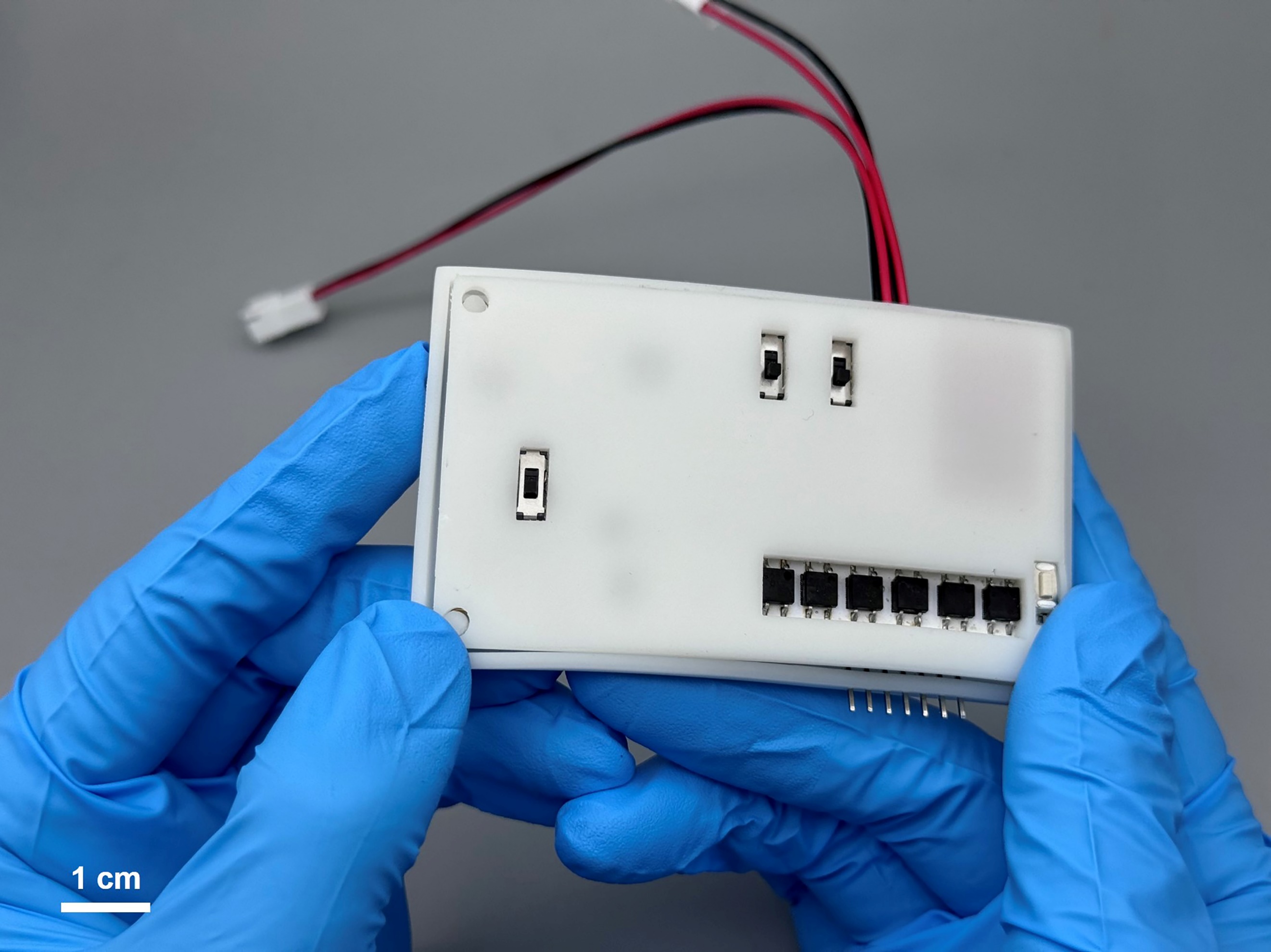


**Fig. S19** The image of a coding circuit with the protective shell


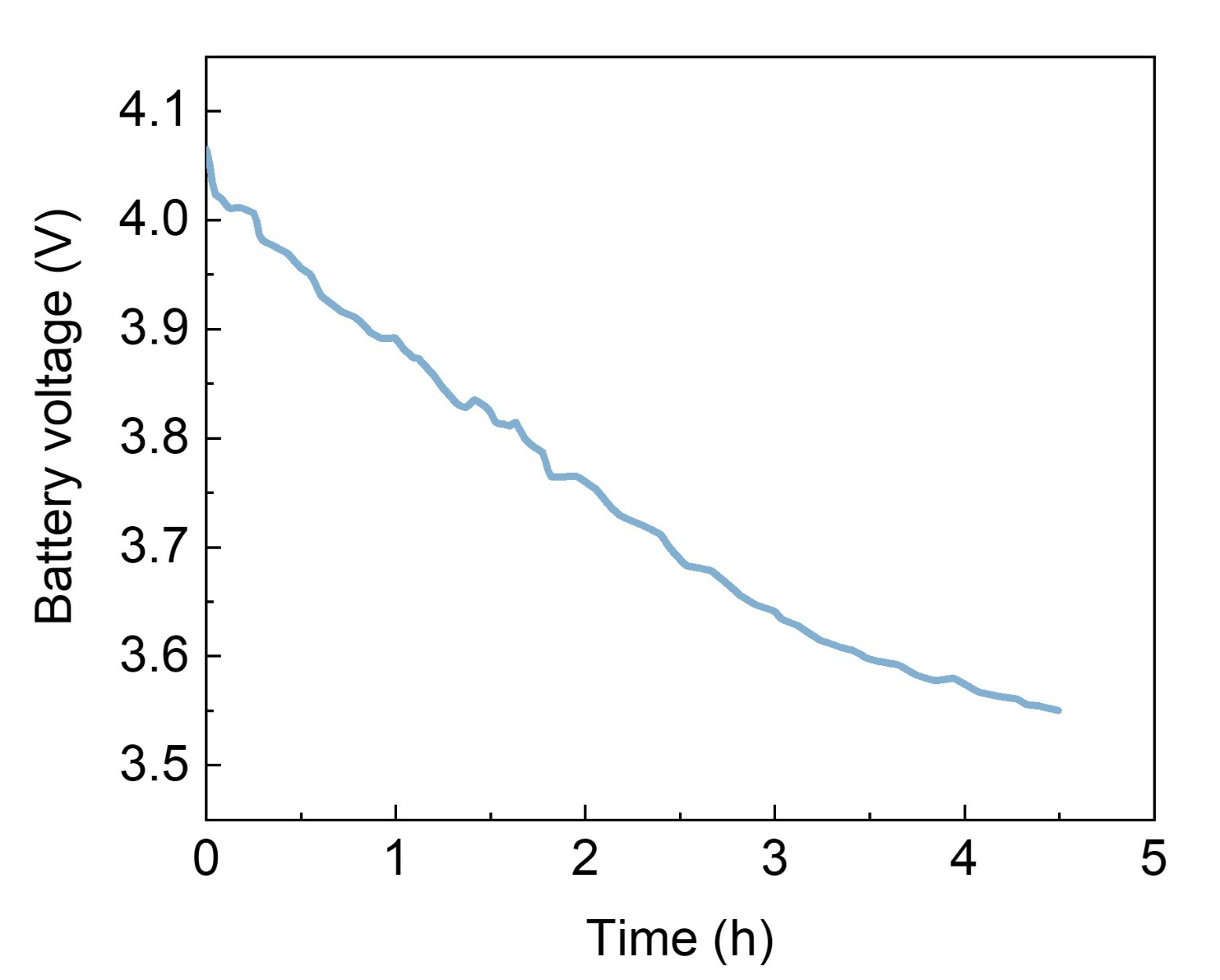


**Fig. S20** Operating time of flexible boost circuit under a full-load state


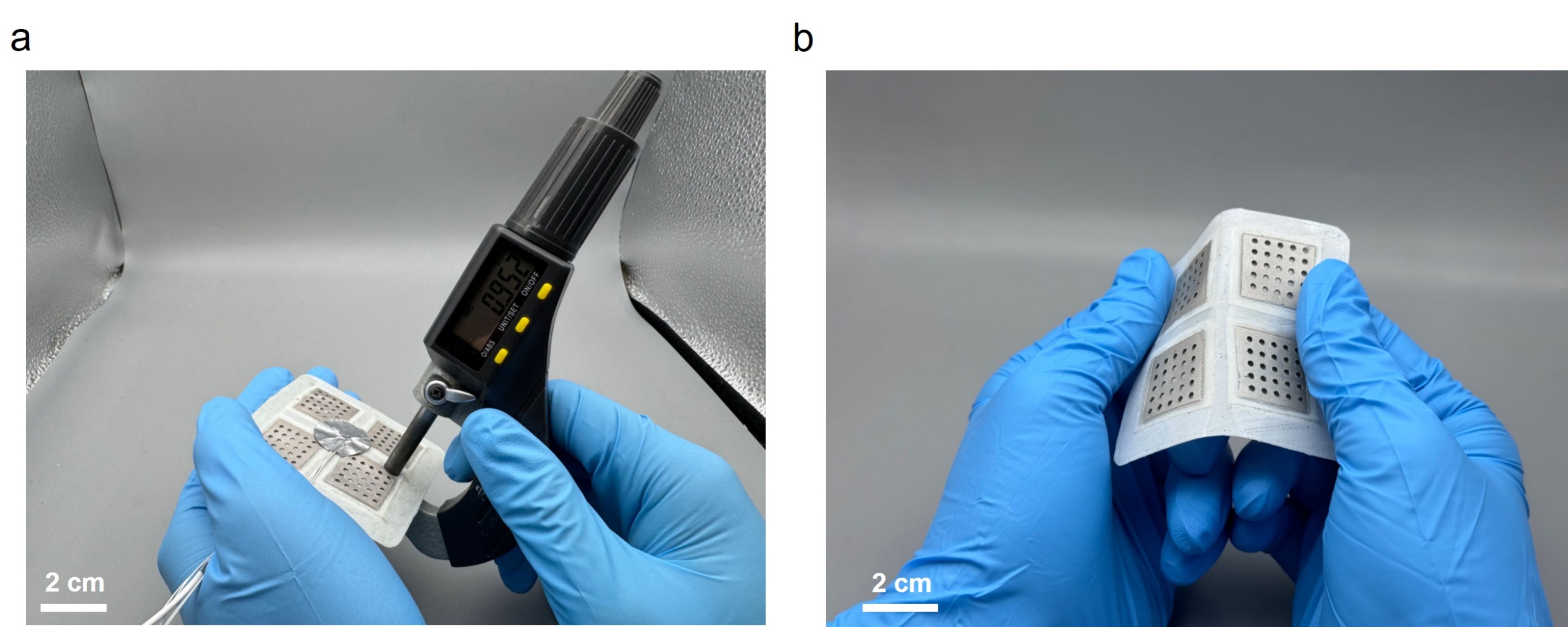


**Fig S21** The images of the 2×2 breeze-sense generators array, each unit is 2×2 cm^2^ with **a** a thickness of ~ 950 µm, and the flexibility of the array is shown in **b**


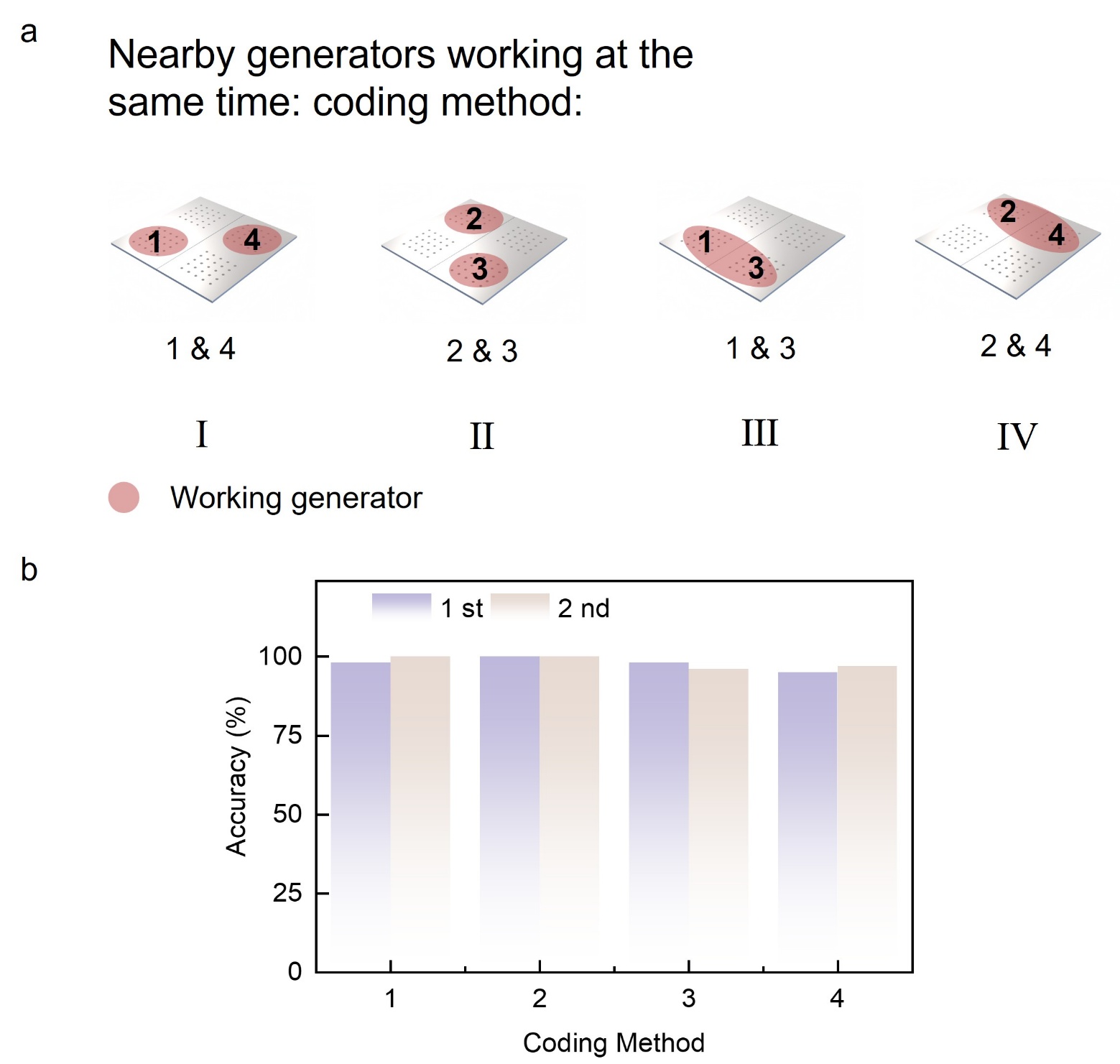


**Fig. S22 a** Schematic of nearby generators working at the same time, and **b** accuracy of volunteers’ test results


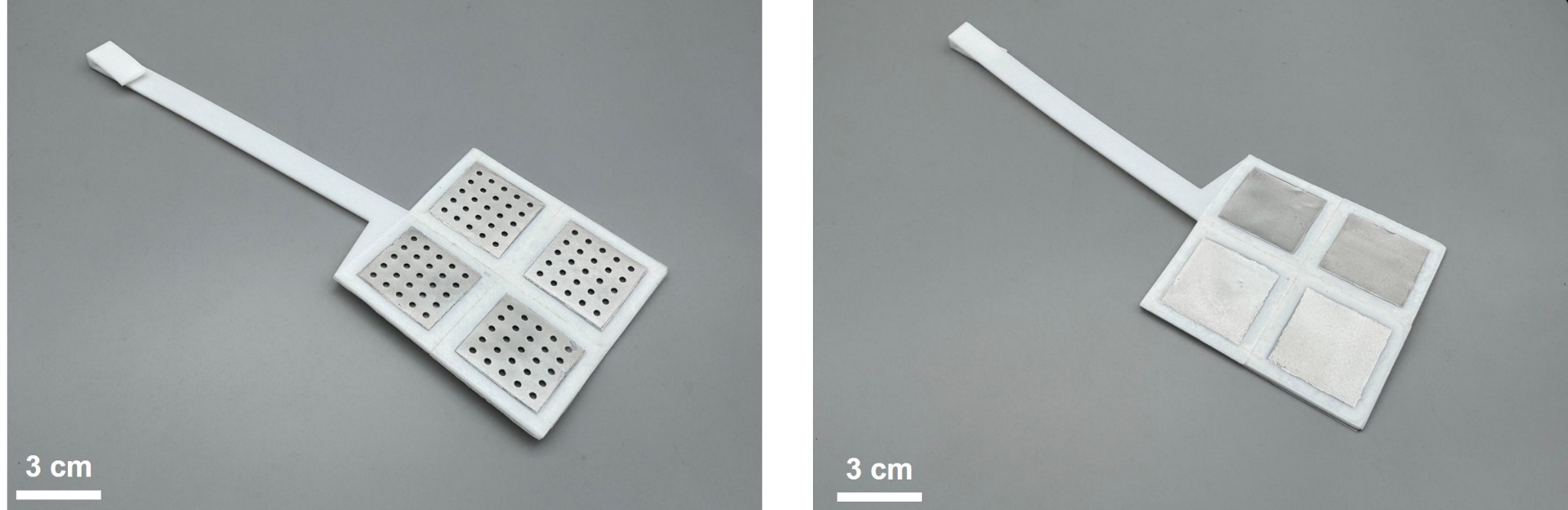


**Fig. S23** The images of front and back sides of a 2×2 breeze-sense generators array, each unit is 3×3 cm^2^


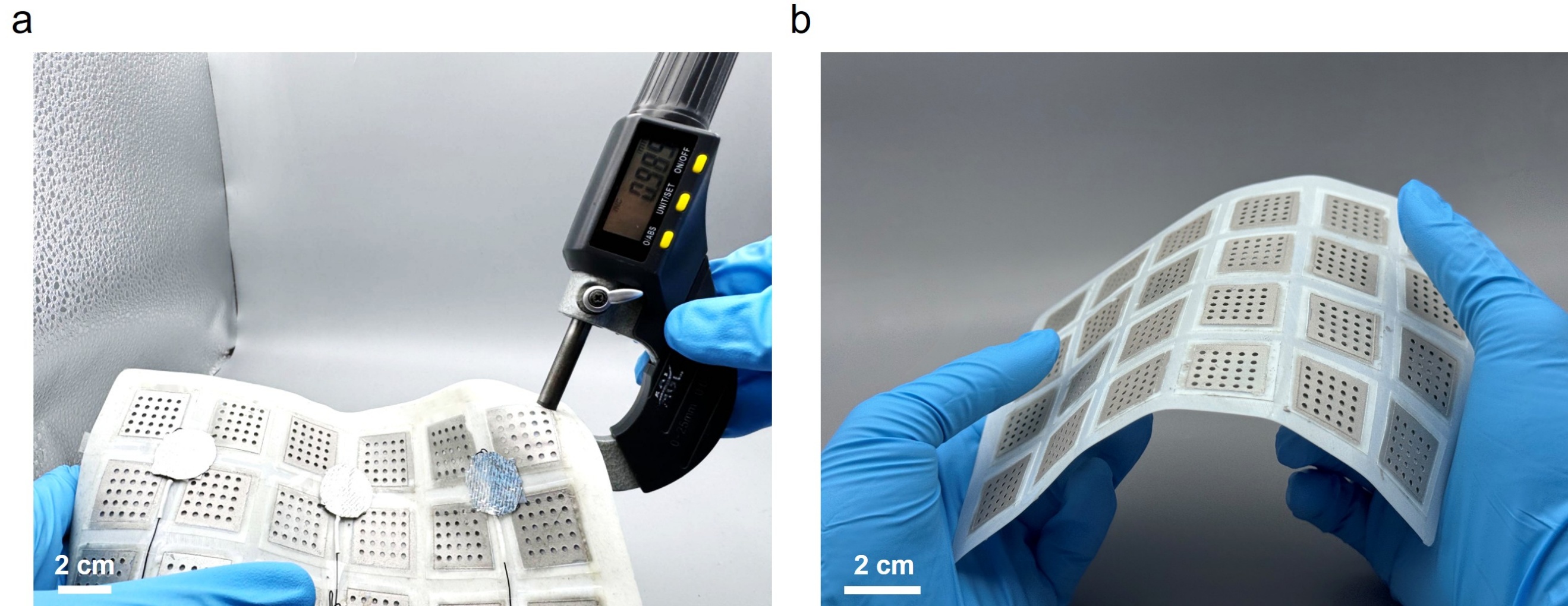


**Fig. S24** The images of the 4×6 breeze-sense generators array, each unit is 2×2 cm^2^ with **a** a thickness of ~ 990 µm, and the flexibility of the array is shown in **b**

**Table S1** The geometrical structure parameters of the breeze-sense generator in the COMSOL simulation


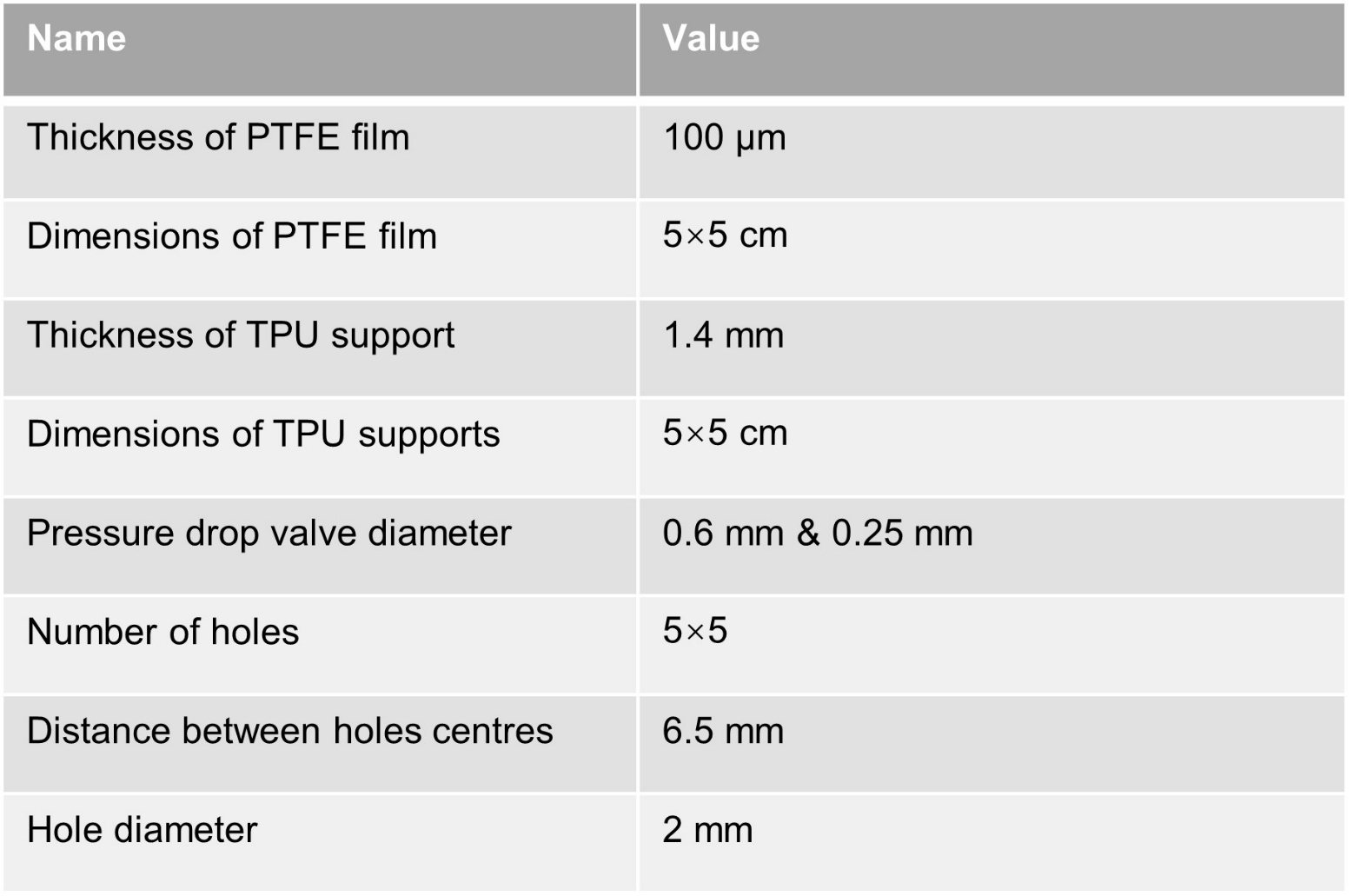


**Table S2** The mechanical characterizations of materials in the COMSOL simulation


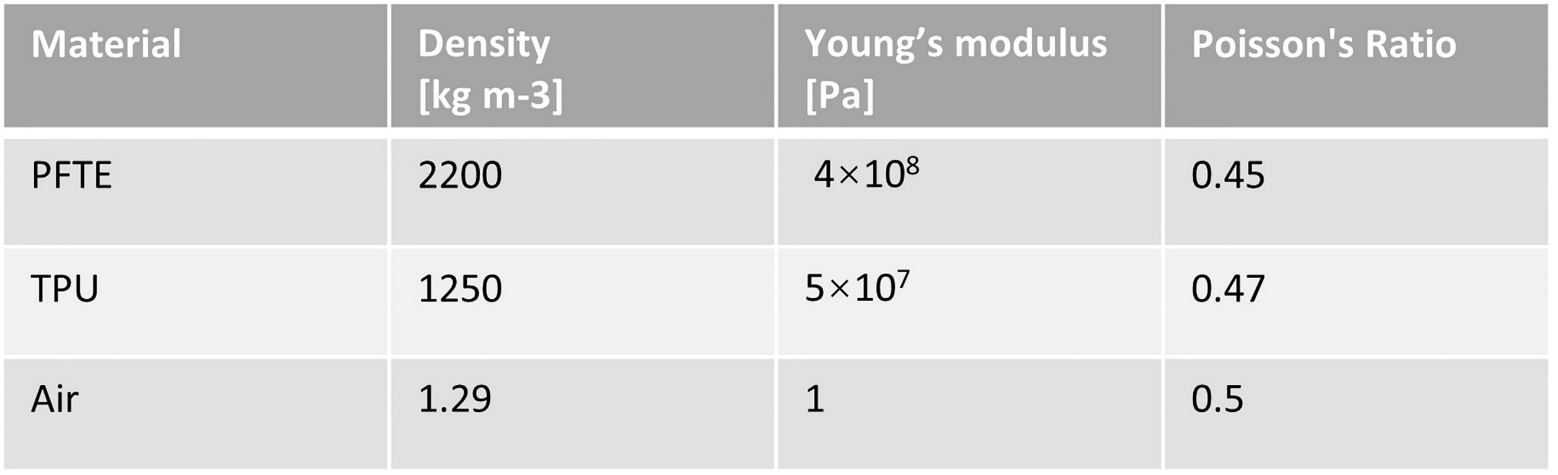


**Table S3** Comparison of key parameters among the non-contact haptic feedback devices


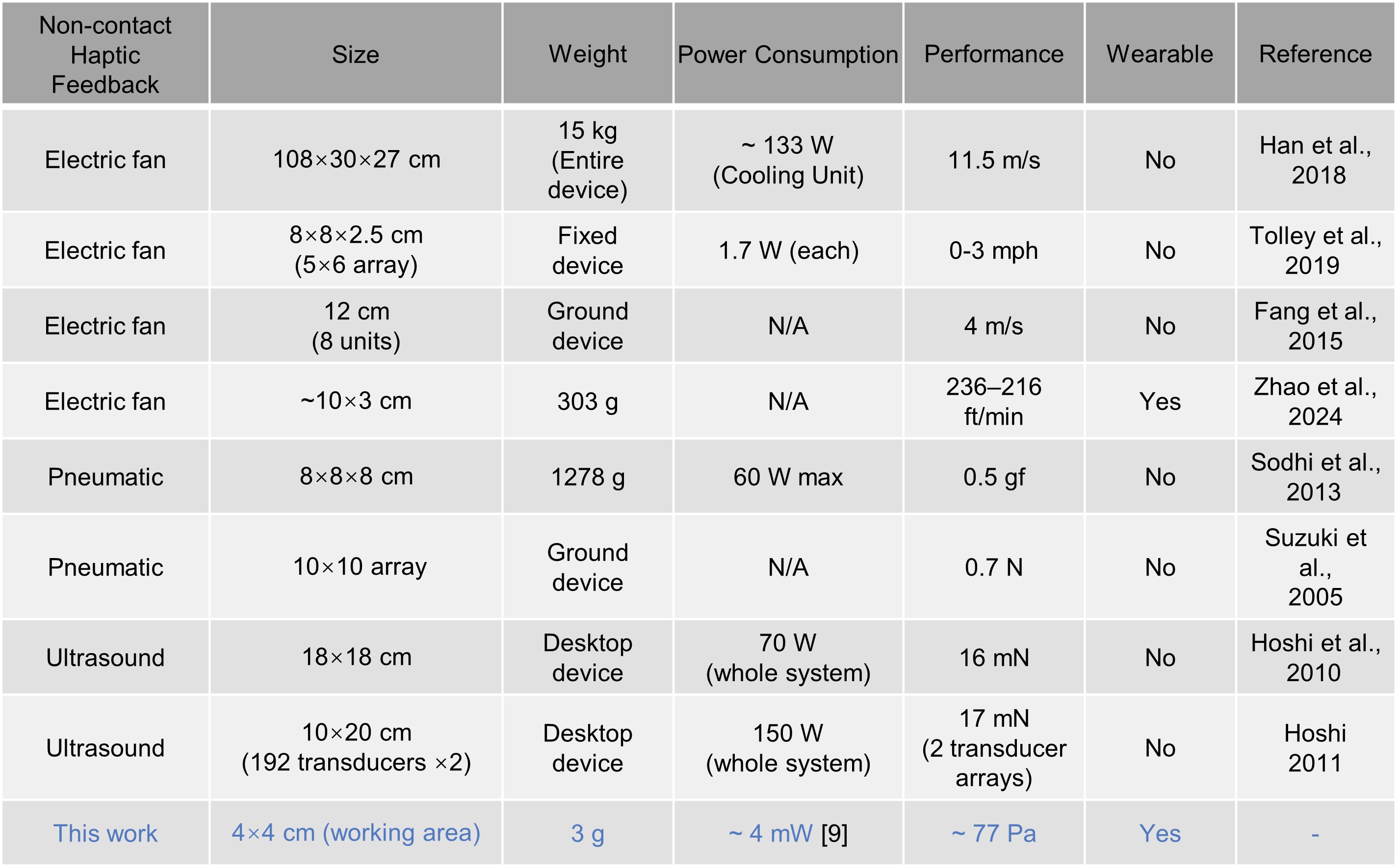


The power consumption of our breeze-sense generator (~ 4 mW) is calculated by employing the values of static capacitance (~ 20 pF) and the phase difference between the driving voltage (1000 V) and current (~ 23.4°), following Equation (S1) [S9].

$P=2\pi fCtan\delta{V_{rms}}^{2}$ (S1)

Compared to other non-contact devices [S1-S8] based on electric fan, pneumatic actuation, and ultrasound, our breeze-sense generator has significant advantages of low power consumption (~ 4 mW) and wearability (3 g, thickness less than 2 mm).

**Supplementary References**

1. P. Han, Y. Chen, K. Lee, H. Wang, C. Hsieh et al., Haptic around: Multiple tactile sensations for immersive environment and interaction in virtual reality. Proceedings of the 24th ACM symposium on virtual reality software and technology (2018), pp.1-10. <https://dl.acm.org/doi/pdf/10.1145/3281505.3281507>
2. D. Tolley, TN. Nguyen, A. Tang, N. Ranasinghe, K. Kawauchi et al., Windywall: exploring creative wind simulations. Proceedings of the Thirteenth International Conference on Tangible, Embedded, and Embodied Interaction (2019), pp.635-644. <https://dl.acm.org/doi/pdf/10.1145/3294109.3295624>
3. M. Feng, R.W. Lindeman, H. Abdel-Moati, J.C. Lindeman, Haptic ChairIO: A system to study the effect of wind and floor vibration feedback on spatial orientation in VEs. 2015 IEEE Symposium on 3D User Interfaces. (2015), pp.149-150. <https://ieeexplore.ieee.org/stamp/stamp.jsp?tp=&arnumber=7131744&tag=1>
4. F. Zhao, Z. Li, Y. Luo, Y. Li, H.N. Liang, AirWhisper: enhancing virtual reality experience via visual-airflow multimodal feedback. J. Multimodal User Interfaces. (2024), pp.1-16. <https://link.springer.com/article/10.1007/s12193-024-00438-9>
5. R. Sodhi, I. Poupyrev, M. Glisson, A. Israr, AIREAL: interactive tactile experiences in free air. AIREAL: interactive tactile experiences in free air. ACM Trans. Graph. **32**, 1-10 (2013). <https://dl.acm.org/doi/abs/10.1145/2461912.2462007>
6. Y. Suzuki, M. Kobayashi, Air jet driven force feedback in virtual reality. IEEE Comput. Graph. Appl. **25**, 44-47 (2005). <https://ieeexplore.ieee.org/abstract/document/1381224>
7. T. Hoshi, M. Takahashi, T. Iwamoto, H. Shinoda, Noncontact tactile display based on radiation pressure of airborne ultrasound. IEEE Trans. Haptics. **3**, 155-156 (2010). <https://ieeexplore.ieee.org/abstract/document/5406524>
8. T. Hoshi, Development of aerial-input and aerial-tactile-feedback system. 2011 IEEE World Haptics Conference. (2011), pp. 569-573. <https://ieeexplore.ieee.org/abstract/document/5945548>
9. T. Jordan, Z. Ounaies, J. Tripp, P. Tcheng, Electrical properties and power considerations of a piezoelectric actuator. MRS Online Proceedings Library (1999). <https://www.cambridge.org/core/journals/mrs-online-proceedings-library-archive/article/abs/electrical-properties-and-power-considerations-of-a-piezoelectric-actuator/4338A69565E567441BF1C4B5148A000D>

**Legends for Movie S1 to S5**

**Movie S1:** Breeze-sense generator blowing a slip of paper

**Movie S2:** Volunteer Test 1: Sequential working modes

**Movie S3:** Volunteer Test 2: Nearby working modes

**Movie S4:** Feel the direction of continuous breeze in virtual reality

**Movie S5:** Feel the sudden breeze in virtual reality
